# Analysis of the Transcriptomes Downstream of Eyeless and the Hedgehog, Decapentaplegic and Notch Signaling Pathways in *Drosophila melanogaster*

**DOI:** 10.1371/journal.pone.0044583

**Published:** 2012-08-31

**Authors:** Landry E. Nfonsam, Carlos Cano, Joann Mudge, Faye D. Schilkey, Jennifer Curtiss

**Affiliations:** 1 Department of Biology, New Mexico State University, Las Cruces, New Mexico, United States of America; 2 National Center for Genome Resources, Santa Fe, New Mexico, United States of America; University of Washington, United States of America

## Abstract

Tissue-specific transcription factors are thought to cooperate with signaling pathways to promote patterned tissue specification, in part by co-regulating transcription. The *Drosophila melanogaster* Pax6 homolog Eyeless forms a complex, incompletely understood regulatory network with the Hedgehog, Decapentaplegic and Notch signaling pathways to control eye-specific gene expression. We report a combinatorial approach, including mRNAseq and microarray analyses, to identify targets co-regulated by Eyeless and Hedgehog, Decapentaplegic or Notch. Multiple analyses suggest that the transcriptomes resulting from co-misexpression of Eyeless+signaling factors provide a more complete picture of eye development compared to previous efforts involving Eyeless alone: (1) Principal components analysis and two-way hierarchical clustering revealed that the Eyeless+signaling factor transcriptomes are closer to the eye control transcriptome than when Eyeless is misexpressed alone; (2) more genes are upregulated at least three-fold in response to Eyeless+signaling factors compared to Eyeless alone; (3) based on gene ontology analysis, the genes upregulated in response to Eyeless+signaling factors had a greater diversity of functions compared to Eyeless alone. Through a secondary screen that utilized RNA interference, we show that the predicted gene *CG4721* has a role in eye development. *CG4721* encodes a neprilysin family metalloprotease that is highly up-regulated in response to Eyeless+Notch, confirming the validity of our approach. Given the similarity between *D. melanogaster* and vertebrate eye development, the large number of novel genes identified as potential targets of Ey+signaling factors will provide novel insights to our understanding of eye development in *D. melanogaster* and humans.

## Introduction

Tissue-specific transcription factors are thought to cooperate with signaling pathways, which function in multiple developmental contexts, to promote patterned expression of tissue-specific target genes [1], [2], [3]. However, the principles governing how transcription factors and signaling pathways interact are not fully understood, in large part because not many targets are known. We are using the *Drosophila* eye as a model to understand how tissue-specific transcription factors and signaling pathways function together to specify tissue development.

One of the major tissue-specific transcription factors involved in eye specification throughout metazoa is the Pax6 paired-homeodomain protein [4]. Consistent with its role in *Drosophila* eye specification, the *Drosophila* Pax6 homolog *ey* is both required for eye development [5], and capable of converting antennal, leg and wing precursors to an eye fate when misexpressed [6]. Vertebrate Pax6 genes are also required for eye development, and ectopic expression can lead to ectopic eye formation [7], [8], [9], [10], [11], [12], [13], [14], [15], [16].

In principle, knowledge of Pax6 transcription factor targets could reveal a lot about the mechanisms by which it promotes eye specification, and recent efforts have identified a number of probable direct Ey targets with functions in *Drosophila* eye development. Four of the five that are currently known also encode transcription factors, including Eyes absent (Eya), Sine oculis (So), Optix and Atonal (Ato) [17], [18], [19], [20], [21], [22], [23], [24], [25]. A few likely direct targets of Eya and So are known, and include *so* itself and *ey*, as well as the genes encoding the Hedgehog ligand [26], the cell cycle regulator String [27], and another transcription factor, Dachshund (Dac) [28]. In addition, a recent effort at identifying Ato targets has offered up some tantalizing candidate targets [29]. However, by and large the genes whose expression is controlled by these transcription factors are unknown. Thus, what happens during “eye specification” remains a black box.

As in other developmental contexts, a number of signaling pathways play important roles in *Drosophila* eye development, including the Hedgehog (Hh), Decapentaplegic (Dpp) and Notch (N) signaling pathways. Hh, Dpp and N signaling function in initiation and maintenance of the morphogenetic furrow, which sweeps across the field of eye precursors during larval and pupal stages, and separates proliferating from differentiating cells [30], [31], [32], [33], [34], [35]. Although the Hh, Dpp and N signaling pathways regulate expression of genes important for eye development, including Ey [36], to our knowledge there are no studies that have attempted to identify additional targets, direct or indirect, of these signaling pathways in the context of eye development.

Considerable evidence suggests that Ey functions in concert with signaling pathways to promote eye development. For instance, differentiating ectopic eye tissues are induced by *ey* misexpression only in wing precursors that lie within or close to regions expressing Dpp and/or Hh, while co-misexpression of Ey with Dpp and/or Hh leads to an expansion in the area of ectopic eye tissue that forms [18], [37]. One mechanism by which Ey could interact with signaling pathways during eye development is through co-regulation of eye gene transcription.

We reason that identification of genes whose transcription is co-regulated by Ey and by Hh, Dpp or N signaling, directly or indirectly, will provide a better understanding of the events that occur during “eye specification”, as well as a more comprehensive understanding of the regulatory network responsible for eye development. Thus, we report a combinatorial approach to identifying targets of Ey and Hh, Dpp or N. We are using Illumina whole transcriptome mRNA sequencing (mRNASeq) and Agilent 4×44 k whole genome expression arrays to dissect the *Drosophila* eye gene network and identify genes that are co-regulated by Ey and/or by the Dpp, Hh or N signaling pathways.

Our mRNASeq analyses have revealed that 2,841 genes are up-regulated at least 3-fold in wing precursors across 7 different genotypes investigated (*ap>ey*, *ap>hh*, *ap>dpp*, *ap>N*, *ap>ey+hh*, *ap>ey+dpp*, *ap>ey+N*); 341 of these genes were validated by Agilent array. Unsupervised principal component analysis (PCA) and 2-way hierarchical clustering analysis suggests that coexpression of Ey+Hh in the wing disc activates expression of genes in a pattern closest to that of a wild-type eye. Analysis of Gene Ontology data reveals that Ey functions together with the signaling pathways to activate expression of genes previously known to be important for eye development, as well as of genes with previously determined roles in neural differentiation and function, but for which a role in eye development has not previously been described. However, most of the candidate targets have unknown functions.

Finally, we have shown that the predicted gene *CG4721*, which was identified by our transcriptomics approach, has a previously unknown function in *Drosophila* eye development. *CG4721* encodes a member of the neprilysin family of metalloproteases. Identification of novel genes involved in *Drosophila* eye development will enhance our understanding of the regulatory network existing between the eye transcription factors and signaling pathways. Given that the *Drosophila* eye regulatory network is at least partially conserved in vertebrates, understanding of this network will have implications in human health and disease treatment.

## Results

### Co-expression of Ey+Hh, Dpp, or N results in large ectopic eye fields in the wing disc

The goal of our study is to use a transcriptomic approach to identify targets co-regulated by Ey and/or by the Dpp, Hh or N signaling pathways. Our first step was to generate a set of tissues for transcriptomic analysis that would likely lead to identification of these targets. One approach would be to compare the wild-type transcriptome to those of loss-of-function mutant tissues for *ey* and/or *dpp*, *hh* or *Notch*. However, *ey* loss-of-function phenotypes range from a small eye to a complete loss of eye and other head precursors [5], [20], [38], [39], [40], [41], [42] making the isolation of mutant tissue problematic. Loss of Hh, Dpp or N signaling in eye-specific or temperature-sensitive alleles likewise results in loss of all or part of the eye [34], [43], [44], [45], [46], [47], [48], [49]. Therefore, a gain-of-function approach was adopted using the *Gal4-UAS* system [50].

**Figure 1:**
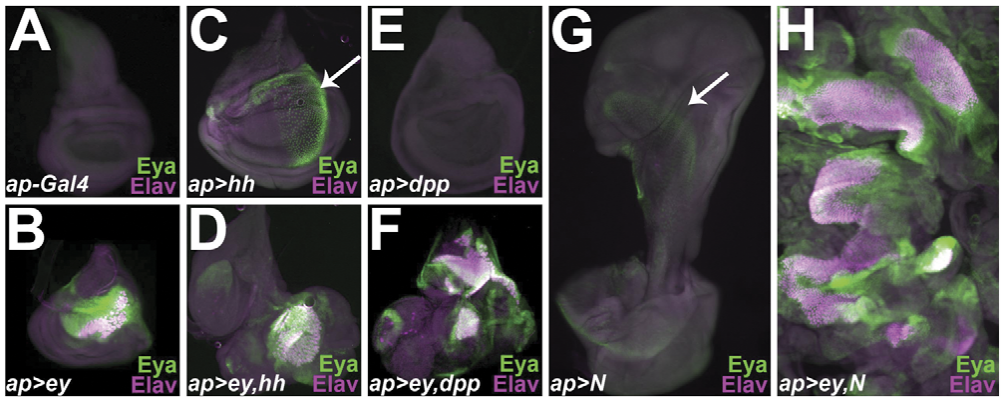
Co-misexpression of Ey together with Hh, Dpp or activated Notch results in larger ectopic eye fields that misexpression of Ey alone. (A–H) Wing discs from third instar larvae of the indicated genotypes stained with anti-Eya and anti-Elav.

We chose the *apterous-Gal4* (*ap-Gal4*) driver [51], [52] to drive expression of *ey* and/or *hh*, *dpp*, or *N* in the dorsal compartment of the wing disc, largely because it drives expression in a large percentage of wing disc cells. To determine the extent of ectopic eye tissue produced, we stained third instar wing discs with antibodies against the neuronal cell marker Elav, which is expressed in differentiating neurons including photoreceptor cells. Wing discs were also stained with anti-Eya, which is expressed in the eye portion of third instar eye-antennal discs in undifferentiated pre-proneural eye precursors anterior to the MF, as well as in cells within the MF and in differentiating eye cells posterior to the furrow [17], making it a suitable marker for both differentiating and undifferentiated eye precursors. The images in Figure 1 represent wing discs from different genotypes. The phenotypes of discs from a single genotype (we have dissected hundreds of discs from each genotype) are remarkably consistent in terms of size, shape, and extent of transformation to eye tissue. In addition, we emphasize that the images of the discs were all taken at the same magnification, and that the images include ***only*** wing disc tissue.

Whereas neither Elav nor Eya is expressed in wild-type wing discs at this stage in development (Fig 1A), a small cluster (∼15% of disc surface area) of differentiating ectopic ommatidia, marked by Elav expression, form in the wing disc as a result of misexpression of *ey* under *ap-Gal4* control (Fig 1B). These ectopic ommatidia also express Eya and are surrounded by cells expressing Eya. The presence of wing disc cells expressing Eya but not Elav indicates that wing tissues are being transformed into eye tissues.

Consistent with their known roles in regulating cell growth and proliferation, misexpression of *hh, dpp* or *N* under *ap-Gal4* control (*ap>hh*, *ap>dpp, ap>N*) results in a larger wing disc, particularly in the case of *ap>N* (Fig 1C,E,G). No ectopic photoreceptors expressing Elav or Eya form in the *ap>hh, ap>dpp* or *ap>N* wing discs, although a few cells in the peripodial membrane of *ap>hh* and *ap>N* discs express Eya (arrows in Fig 1C,G).

In contrast, co-expression of *ey+hh*, *ey+dpp* or *ey+N* under *ap-Gal4* control (*ap>ey,hh*; *ap>ey,dpp* and *ap>ey,N*) results in enlarged wing discs containing a larger field (30–40% disc surface area) of differentiating (Elav + Eya-expressing) ectopic eye precursors, and an even larger field of undifferentiated (Eya-expressing) ectopic eye precursors, compared to misexpression of *ey* alone (Fig 1D,F,H). The strongest response was observed for *ap>ey,N* with complete distortion of the wing disc, accompanied by multiple large areas of differentiating ectopic eye tissue. Thus, as has been previously shown [18], [37], co-misexpression of Ey and signaling pathways important for eye development appears to be more efficient at directing eye development than Ey alone.

### Generating transcriptomes downstream of Ey+signaling pathways using mRNASeq

To identify genes whose transcription is co-regulated by Ey and/or signaling pathways that promote eye development, we used the Illumina Genome Analyzer II (GAII) platform (sequencing-by-synthesis and Reverse Termination; Illumina, Inc., San Diego, CA) to profile the transcriptome resulting from ectopic expression of *ey*, *hh*, *dpp*, *N*, *ey+hh*, *ey+dpp*, or *ey+N* in the *Drosophila* wing disc. Control eye-antennal discs and control wing discs from the *ap-Gal4* strain, and wing discs misexpressing Ey and/or the different signaling molecules were dissected and mRNA purified from each pooled genotype (150 discs pooled from each genotype).

cDNA libraries were generated from purified mRNA and Illumina-sequenced at the National Center for Genome Resources (NCGR) in Santa Fe, New Mexico, as previously described [53], [54], [55]. An average of ∼11±1.8 million high quality reads [average PHRED score of 30 = 99.9% accuracy [56]] of 36 bp length was generated per genotype for an average of ∼396±67.1 Mb per library (Table 1).

**Table 1:** Summary of mRNAseq results.

Sample Genotype	Average Read Length	Average Read Quality	Number of Reads	Depth Genome (∼120Mb)	% reads aligned Genome	% reads aligned Transcript	% unique aligned Genome	% unique aligned Transcript	Number Gene Matches	Number Transcript Matches
*ap-Gal4* eye-antennal disc	36	30	6,016,683	2X	88%	84%	86%	43%	11218	18103
*ap>ey*	36	30	14,152,027	4X	85%	85%	84%	45%	11812	18472
*ap>hh*	36	30	16,483,589	4X	87%	83%	85%	43%	12577	19316
*ap>dpp*	36	30	4,265,145	1X	85%	82%	83%	41%	10454	16482
*ap>N*	36	30	4,668,369	1X	86%	84%	85%	43%	11022	17251
*ap>ey,hh*	36	30	4,567,902	1X	86%	84%	83%	45%	10754	17300
*ap>ey,dpp*	36	29	17,133,136	5X	87%	83%	86%	42%	12440	19259
*ap>ey,N*	36	35	14,934,353	4X	81%	74%	79%	37%	11562	18466
*ap-Gal* wing disc	36	30	13,548,924	4X	74%	69%	72%	35%	12048	18576

The sequences were loaded into the Alpheus software developed at NCGR [57] and aligned to the *Drosophila* Genomic Sequence Release 5 (Berkeley *Drosophila* Genome Project) and to the All-Transcript Sequence Release 5.21 [58] using the GSNAP algorithm (minimum % alignment: 94% corresponds to a minimum identity count of 34/36).

A large majority of reads aligned to both genome and transcriptome: an average of 84±1.5% of reads aligned to the *Drosophila* genome, with 83±1.5% aligning uniquely, and an average of 81±1.8% reads aligned to the transcriptome, with 42±1.1% aligning uniquely. Reads that showed no alignment could be contaminants, low quality reads or quality reads generated from non-annotated regions. The fact that the percentage of reads aligning to the transcriptome is similar to the percentage aligning to the genome confirms that most *Drosophila* genes have been annotated. The significantly lower percentage of uniquely aligned reads in the transcriptome versus the genome most likely reflects alignment of reads to common regions of multiple alternative transcripts.

The number of genes expressed in each library was very large: an average of 86±1.8% of annotated *Drosophila* genes were represented by at least one read, with 83±1.5% of known *Drosophila* transcripts having at least one read. Table 1 summarizes the data described above for each of the genotypes analyzed.

For the analysis described below, we focused on genes having at least one uniquely genome-aligned read. For each genotype, read abundances for individual *Drosophila* genes were quantified by direct counts of reads aligned to specific genomic loci, normalized as reads per million, as determined by the Alpheus analysis software [57].

### Coverage depth as determined by read abundance

Knowledge about depth of coverage is important in determining confidence about levels of gene expression in cDNA libraries, particularly for low abundance transcripts. Deeper coverage also increases the number of reads aligning to specific genomic loci, improving not only the reliability of gene calling but also the chances of identifying genomic variations such as SNPs, alternative splice sites, insertions, deletions and alternative polyA sites.

To approximate the depth of coverage for our cDNA libraries, we first made comparisons to current information about *Drosophila melanogaster* genome and transcriptome sizes. When compared to the total size of the *Drosophila* genome (120Mb) [59], [60], the average depth of coverage for the libraries was ∼3±0.5X. Given an estimated transcriptome size of 50.5 M b [58], the average coverage depth was 7.6±1.3X.

**Figure 2:**
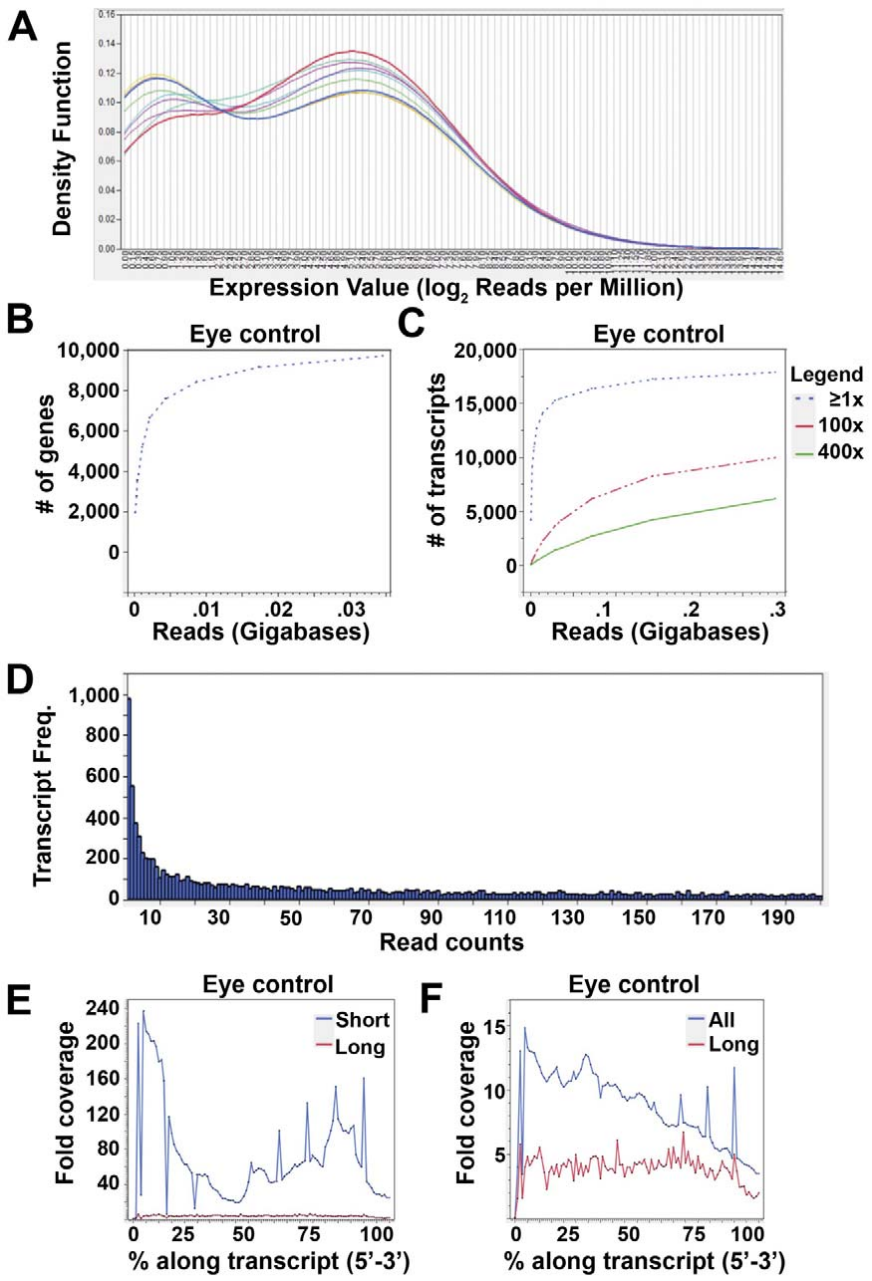
Illumina mRNAseq data are of high quality. (A) Overlaid one-way kernel density distribution curves for the 9 mRNAseq libraries generated, showing strong similarity among the libraries. (B,C) Graphs of the number of genes detected as a function of the amount of sequence data generated for the eye control library for both genome (B) and transcriptome (C) aligned reads, demonstrating the depth of sequence data. (D) Histogram showing number of transcripts plotted versus the number of reads representing each transcript for the eye control library, demonstrating that a large percentage of genes are represented by at least 2 reads. (E,F) Histogram showing read coverage, averaged across all transcripts with aligned reads, calculated at 1% intervals along the 5′ to 3′ extent of each transcript, showing little bias in coverage. “Short” transcripts are ≤500 bp; “long” transcripts are ≥10,000 bp.

Other analyses demonstrated remarkable similarities in read distribution among the libraries. For instance, we generated one-way kernel density distribution curves for the different libraries investigated (Fig 2A). No outliers were detected, and strain curves fell within the limits of the two extreme distributions, the eye and the wing controls, thus confirming that all libraries are of a similar high quality and that similar numbers of genes were detected in all libraries. We further performed a pair-wise sample correlation plot of genomic read abundance to determine the strength of relationship between the different libraries. The pair-wise correlation coefficients (*r*) between libraries were very high (Fig S1), ranging from 0.92 to 0.99. This further confirms the similar distribution pattern of read abundance across all 9 libraries.

Given the similarity among all of the libraries, we focused our next analyses on the eye control library. To determine whether the 11,218 genes (84% of known genes) detected by ≥1 read in the eye control library is a good estimate for the total number of genes expressed in the *Drosophila* eye-antennal disc at the third larval instar, we plotted the number of genes detected as a function of the amount of sequence data generated (Fig 2B). At approximately one million reads (∼35 Mb; ∼17% of total number of reads generated), ∼10,000 known *Drosophila* genes (75%) were detected by at least one read (≥1X), and the curve was approaching a plateau. This result suggests that our sequencing was deep enough to detect most of the genes expressed at the third-instar larval stage of eye development.

A similar curve was observed when number of transcripts detected by ≥1 read in the eye control library (18,103; 83% of known transcripts) was plotted against read abundance (Fig 2C). In addition, we generated transcript curves for 2X, 50X, 100X and 400X coverage by dividing the read abundance by the corresponding fold factors and determining the number of detected transcripts. There were no observable differences in the transcript curves generated at 2X or 50X (data not shown) compared to ≥1X. Changes compared to the ≥1X curve were observed at 100X and at 400X (Fig 2C), indicating that sequencing has to be very deep to observe an appreciable change in the number of detected transcripts with increased read abundance. Together, these data confirm that the depth of sequencing was sufficient to detect even genes expressed at low levels.

We also analyzed the distribution of coverage across the eye control library by generating a histogram showing the number of transcripts represented by particular numbers of reads (Fig 2D). As in other contexts [61], as read abundance (measured in read counts) increased, the number of transcripts declined greater than exponentially (R^2^ = 9.8). Thus, very few transcripts were expressed at levels ≥10,000 reads (≤0.6±0.02%), and only ∼4.6±0.01% were expressed at ≥2,000 reads. Nevertheless, of the 18,103 expressed transcripts, only 5.4% were represented by only one read. 59.7±0.03% of transcripts were represented by 1–200 reads, and 40±0.0005% of transcripts had an abundance of ≥200 reads. Thus, more than 90% of detected transcripts are represented by at least 2 reads.

Finally, a curve of read abundance along transcript length was determined for the eye control library at 1% intervals from the 5′ to 3′ end (Fig 2E,F). These data showed that read abundances are reasonably randomly distributed, particularly for long transcripts. The decrease in read abundance at both the extreme 5′ and 3′ termini is suggestive of “edge effects”: of random hexamers not aligning properly to sequences very close to the ends of cDNAs. Although a slight bias towards the 3′ end might have been expected given that transcripts were polyA mRNA enriched (see Methods), a mild bias was in fact observed towards both the 5′ and 3′ end for short transcripts (Fig 2E) and towards the 5′ end of all transcripts (Fig 2F). This may be attributed to some inherent bias of the random hexamers towards the 5′ end during cDNA priming. However, the effect was so mild that we do not expect it to affect our interpretations below.

Based on the results described above, our mRNASeq data appears to be of high quality and to provide sufficient depth of coverage to allow for detailed interrogation of the *Drosophila melanogaster* transcriptome. At such high quality, the mRNASeq data provides suitable information with which to identify novel eye-specific genes co-regulated by *ey* and the *hh*, *dpp* or *N* signaling pathways in a precise and reliable manner.

### Agilent array analyses of *D. melanogaster* genome

To enable validation of the results of our mRNASeq data with respect to individual genes of interest, we performed one-color microarray analyses for each of the 9 genotypes used for mRNASeq. We prepared mRNA from wing and eye-antennal discs from third instar larvae as in the case of mRNASeq, except that in this case we prepared 4 replicates of mRNA for each genotype. All 36 labeled cDNA libraries generated from purified mRNA were hybridized to *D. melanogaster* whole genome 4X44K Agilent expression arrays. Probe intensities extracted from image data were normalized using the Quantile Normalization package in “R” [62].

As with the mRNASeq data, a number of tests found no outliers among the different genotypes for the array data. We generated kernel density log_2_ transformed distribution curves of Quantile Normalized array data (averages across the four replicates for each of the nine genotypes). No outliers were detected, and the distribution pattern was similar to that observed for the mRNASeq data (Fig S2A). However, the array data were even more similar across all nine genotypes compared to that for the mRNASeq data. Great similarities across the nine genotypes were also observed with pair-wise sample correlations, with pair-wise correlation coefficients ranging between 0.94 and 0.99 (Fig S3). Box plots and Relative Log Expression plots gave similar results (Fig S2B,C). The above analyses suggest that our array data is of very high quality.

To identify differentially expressed genes among the different genotypes, we carried out a SAM statistical analysis [63] using the q-value method developed by Storey and Tibshirani [64]. The analysis utilized a two-class unpaired analysis to compare expression levels between two genotypes. Differences between two genotypes are expressed as fold changes.

### Comparison of Agilent Array to RNASeq data

To compare array and mRNAseq data, for the eye control we generated a scatter plot of array log2 transformed, average fold change intensities (average intensities of all 4 normalized replicates relative to the wing control) versus mRNASeq log2 transformed, fold change reads per million (relative to the wing control) (Fig S4). As observed in previous studies [61], the calculated correlation coefficient (R^2^ = 0.4) showed a weak relationship between the two data types. However, as has also been shown by others [61], mRNASeq data showed a wider dynamic range of 1.2 orders of magnitude greater than that for the array data (log_2_ dynamic range RNASeq-19.8; log_2_ dynamic range array hybridization-14.4).

Moreover, 4,425 genes across all genotypes (excluding the wing control) were identified as being ≥3-fold up-regulated relative to the wing control by mRNAseq, compared to 1,253 genes identified by Agilent array. Thus, in accord with other studies, our results suggest that the mRNAseq approach is much more sensitive at detecting changes in gene expression.

### Ey+signaling factor transcriptomes are closer to the eye control than Ey alone

Misexpression of Ey+signaling factors important for eye development results in larger patches of ectopic eye tissue in wing discs compared to Ey alone (Fig 1) [18], [37]. We hypothesize that this difference occurs because some genes important for eye development are co-regulated by Ey and signaling factors, as opposed to Ey alone. However, it is also possible that the expression of the signaling factors leads to more tissue that can be acted upon by Ey, resulting in a larger ectopic eye.

**Figure 3:**
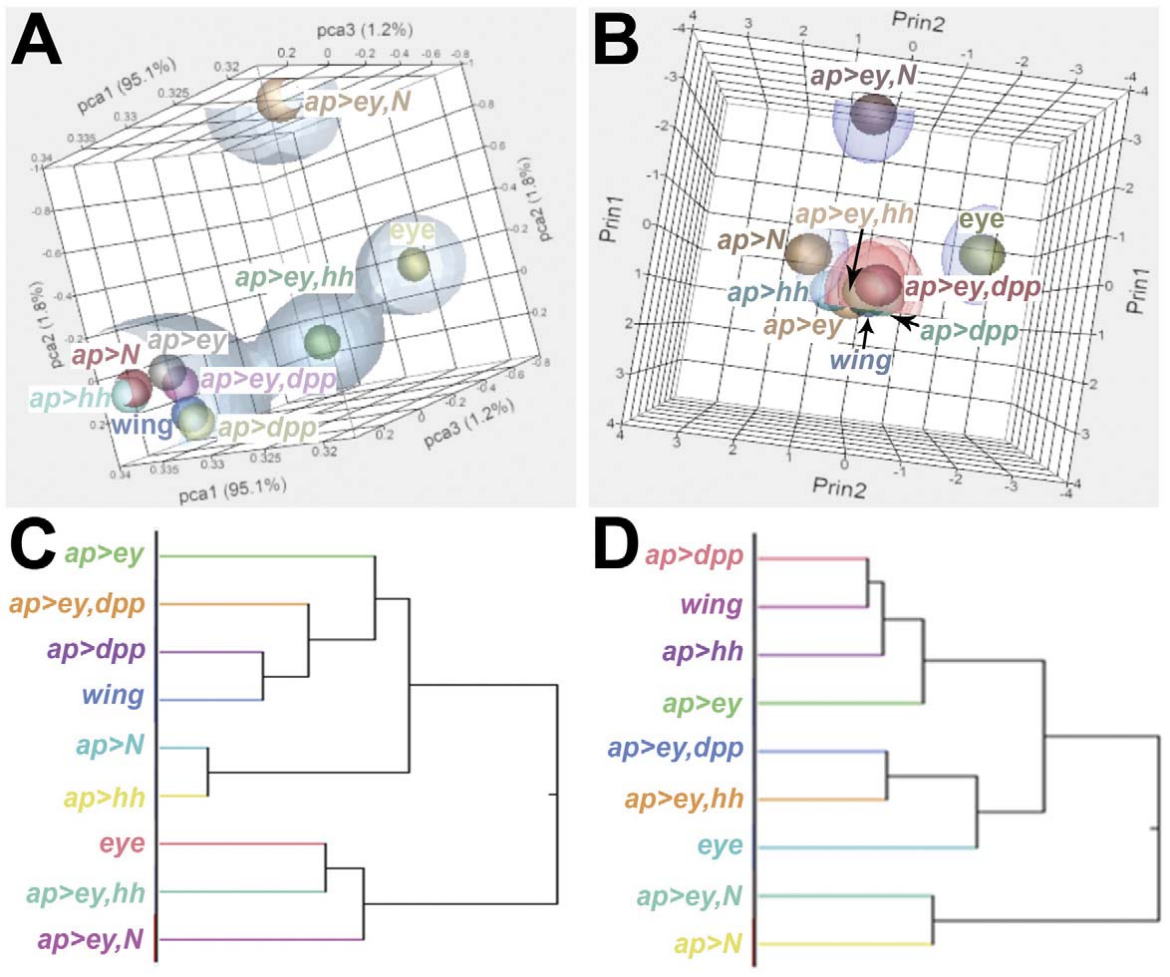
The transcriptome resulting from Ey+Hh misexpression clusters more closely with the eye control than does the Ey transcriptome. (A,B) Unsupervised principle component analysis and (C,D) 2-way hierarchichal clustering analysis. (A,C) mRNASeq data and (B,D) array data.

If Ey and the signaling pathways co-regulate genes important for eye development, then the pattern of expression in the Ey+signaling factor transcriptomes should be closer to the eye control transcriptome than the Ey-only transcriptome is. To test this we performed unsupervised principal component analyses (by Pearson product-moment correlation) and 2-way hierarchical clustering analyses on the mRNASeq (genome-aligned reads per million; log_2_ transformed) and the array transcriptomes (Quantile Normalized signal intensities; log_2_ transformed) (Fig 3).

Although there are some differences in the pattern of clustering between the mRNASeq and array data, one consistent result is that the *ap>ey,hh* transcriptome clusters closer to the eye control than does the *ap>ey* transcriptome. These results provide support for the hypothesis that Ey and the Hh signaling pathway co-regulate transcription of genes important for eye development.

### Transcriptomic analyses reveal appropriate expression changes, plus some surprises

**Figure 4:**
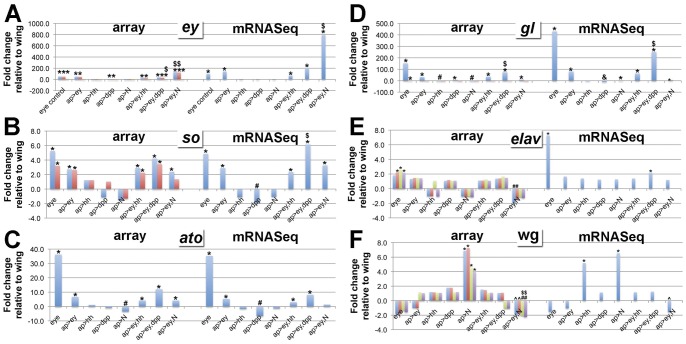
Eye genes are upregulated when Ey+signaling factors are misexpressed in the wing disc. Graphs show fold change values for array and for mRNAseq for genes associated with eye development or with wing development for different libraries relative to the wing control. Array data: values are averages for all 4 replicates; different probes present on the arrays are shown in different colors; “*” and “#” indicate statistically significant upregulation or downregulation, respectively; “$” indicates statistically signficant upregulation in *ap>ey*+signaling factor versus both *ap>ey* and *ap>*signaling factor; “?” indicates statistically significant upregulation in *ap>ey*+signaling factor versus either *ap>ey* or *ap>*signaling factor; in all cases statistical significance was determined by SAM analysis. mRNASeq data: based on genome-aligned unique reads; “*” and “#” indicate fold changes versus wing that is greater than 1.7X or less than −2.5X , respectively, as determined in Figure 4; “$” indicates fold change greater than 1.7X in *ap>ey*+signaling factor versus both *ap>ey* and *ap>*signaling factor; “?” indicates fold change greater than 1.7 in *ap>ey*+signaling factor versus either *ap>ey* or *ap>*signaling factor; “&” indicates 0 reads aligned to a particular gene, with fold changes determined using 0.5 to avoid division by 0.

To further assess the reliability of our mRNASeq and array data for downstream analyses, as well as to potentially make discoveries about the relationships between genes already known to have roles in eye development, we examined fold changes in several known genes in wing discs misexpressing *ey*, *hh*, *dpp*, *Notch* and the various combinations relative to the wing control (Fig. 4; Table S1). In the case of both mRNASeq and array, we focused exclusively on normalized data (reads per million for mRNASeq data; Quantile Normalization for array data). Thus, it is possible to compare fold changes across all libraries. Genes included in our analyses were the following:

1. Factors misexpressed in the wing discs used to generate the libraries (*ey*, *hh*, *dpp*, and *Notch*), which we expected to be upregulated under *ap-Gal4* control.
2. Factors whose expression we did not expect to change much, including *twin of eyeless* (*toy*), which encodes a Pax6 homolog that functions upstream of *ey* in eye development [65], as well as *homothorax* (*hth*) and *teashirt* (*tsh*), which are involved in development of both the eye-antennal and the wing disc (c.f. [66], [67], [68], [69]).
3. Transcription factors known to function downstream of Ey for eye fate specification: *eyes absent* (*eya*), *sine oculis* (*so*), *dachshund* (*dac*), *distal antenna* (*dan*) and *distal antenna related* (*danr*), as well as other genes that are thought to be direct targets of Ey: *shifted* (*shf*), *Optix*, *atonal (ato)* [23], [24], [25], [65], [70], [71], [72], which we expected to be upregulated in the wing control under direct or indirect control of Ey.
4. *elav*, whose protein product is known to be upregulated in the wing as a result of Ey or Ey+signaling factor expression [18], [37] (Fig 1).
5. Genes with known functions in eye development that we identified because they were upregulated in the wing disc at very high levels in response to *ap>ey*+signaling factors: *glass* (*gl*), *scratch* (*scrt*), *sevenless* (*sev*), *lozenge* (*lz*), *prospero* (*pros*) and *ocelliless/orthodenticle* (*oc*) [22], [41], [73], [74], [75], [76], [77], [78], [79], [80], [81], [82], [83].
6. Genes with functions in wing disc but not eye-antennal disc patterning: *vestigial* (*vg*) and *nubbin* (*nub*) (c.f. [84]), which we expected to be higher in the wing control compared to the eye control and to be down-regulated in wing discs in response to *ap>ey*+signaling factors.
7. *wingless* (*wg*), which is a negative regulator of eye development, and which we predicted would be downregulated in wing discs in response to *ap>ey*+signaling factors.

Expression of most of the genes listed above behaved as expected, validating our data. For others there were surprises, as detailed below, which provide exciting hints into the network involved in eye development, but will need to be confirmed experimentally in future work.

### Ey+N synergize to promote ey transcription

As expected, *ey* misexpression in the wing disc under *ap-Gal4* control (*ap>ey*; *ap>ey,hh*; *ap>ey,dpp*; *ap>ey,N*) leads to a significant increase in *ey* levels in the wing disc in both array and mRNASeq data (Fig 4A,*; Table S1), but *ey* levels are not strongly affected in *ap>hh*, *ap>dpp* or *ap>N* wing discs. Interestingly, however, the combination of Ey+N (*ap>ey,N*) activates *ey* transcription to a statistically significantly greater extent than either *ap>ey* alone in both array and mRNASeq data (Fig 4A,$). Thus, there appears to be a positive synergism between Ey and Notch signaling with respect to activating *ey* transcription (see Discussion for possible significance of this result).

### Ey+Hh, Ey+Dpp and Ey+N have different effects on eye- and wing-specific gene expression

Interestingly, for many of the eye- and wing-specific genes, including *eya*, *so*, *ato, dac*, *dan*, *danr*, *gl*, *scrt*, *sev*, *lz*, *pros* and *oc* for the eye, and *vg* and *nub* for the wing, there are differences in the response to Ey+Hh, Ey+Dpp and Ey+N (Fig 4; Table S1). For many of the eye genes, for example *so*, *ato* and *gl* (Fig 4B,C,D), expression in *ap>ey,hh* and *ap>ey,N* wing discs is comparable to or even below levels in *ap>ey*, but expression in *ap>ey,dpp* wing discs trends higher than in *ap>ey*. In part, the reduction observed in *ap>ey,hh* and *ap>ey,N* may be due to the fact that expression of the signaling factors alone often reduces eye gene expression, sometimes significantly (e.g. Fig 4B,#; Table S1). Thus, expression of these genes may be upregulated by Ey misexpression, but independently downregulated by Hh and Notch signaling, resulting in intermediate levels in *ap>ey,hh* and *ap>ey,N* wing discs. In contrast, at least in the subset of genes we examined, Ey+Dpp appear to synergize to raise transcription levels of many eye genes significantly above the levels induced by Ey alone.

As expected, *vg* and *nub* transcription is significantly lower in the eye control versus the wing control in both array and mRNASeq data. However, both genes are only significantly downregulated in wing discs by the Ey+N combination (Table S1). The fact that *vg* and *nub* levels are significantly reduced in *ap>ey,N* compared to *ap>ey* and *ap>N* alone (Table S1) suggests that the Ey+Notch combination is sufficient to reduce their expression. See discussion for the possible significance of these differences in expression of eye- and wing-specific genes in response to different Ey+signaling factor combinations.

### elav

Not all eye genes expected to be upregulated in response to Ey, Ey+hh, Ey+dpp or Ey+N were in fact upregulated. One prominent example is *elav*. Elav protein is not detectable by immunofluorescence in normal wing discs at the stages we harvested tissue for our transcriptome analysis. However, Elav protein is clearly upregulated in the wing in response to misexpression of Ey or Ey+signaling factors (Fig. 1). Surprisingly, however, *elav* transcription is in general not significantly upregulated in *ap>ey*, *ap>ey,hh*, *ap>ey,dpp* or *ap>ey,N*. In fact, two of the array probes show a significant downregulation of *elav* in *ap>ey,N* (Fig 4E,#). One intriguing possible explanation for this data is that the increase observed in Elav protein expression in the wing disc in response to Ey is due to post-transcriptional regulation (see Discussion).

### *wg* is downregulated by Ey+N

High Notch activity along the dorsal/ventral compartment boundary in the wing disc is known to activate Wg expression, promoting the formation of sensory bristles along the wing margin [85], [86], [87]. Accordingly, *wg* transcription is significantly elevated when activated Notch is expressed in the dorsal compartment in *ap>N* wing discs (Fig 4F,*; Table S1).

In the eye portion of the eye-antennal disc, *wg* antagonizes eye development and promotes the formation of cuticular structures [88]. Current models suggest that growth of the eye-antennal disc during eye development in response to Notch signaling allows for physical separation of an anterior, eye-inhibitory Wg-expressing domain from a posterior, eye-promoting Dpp-expressing domain, allowing eye development to occur [89], [90]. This model does not require wg transcription to be regulated.

Interestingly, however, we found that *wg* is significantly reduced in *ap>ey,N* wing discs compared to *ap>N* for both array and mRNAseq data (Fig 4F ^,?,^$). For two of the array probes, *wg* is significantly reduced below levels in *ap>ey* and the wing control (Fig 4F,#). Thus, whereas misexpressing activated Notch alone activates *wg* expression as would be expected for wing development, the Ey+N combination inhibits *wg* expression in the wing precursors, which is likely necessary to allow them to develop as eye precursors instead. Future work will show whether the Ey+N combination functions in a similar capacity to regulate *wg* transcription in the eye-antennal disc, and thus whether it is necessary to revise the current model for how *wg* antagonism of eye development is relieved to allow for eye development.

### Summary

Most of the genes examined above have a pattern of expression across the *ap>ey*, *ap>hh*, *ap>dpp*, *ap>N*, *ap>ey,hh*, *ap>ey,dpp* and *ap>ey,N* libraries that is consistent with what is previously known. In addition, there were a few intriguing surprises that will be discussed further below.

### Identification of candidate genes downstream of Ey and/or Hh, Dpp or N

To generate a list of candidate genes most likely to be important for eye-antennal disc development versus wing disc development, we first identified a set of 1,584 genes from the mRNASeq eye control library (genome-aligned, ≥1 read) and a set of 654 genes from the array eye control library that were ≥3 fold upregulated compared to the respective mRNASeq and array wing control libraries. We then generated a list of 503 genes found in common between the two sets. This list constitutes the “eye control” gene list (Table S2). Similarly, we generated a “wing control” gene list composed of 70 genes ≥3 fold upregulated in both mRNASeq and array wing control libraries (Table S2) and a “No Change” list of 6,238 genes whose expression didn't vary between eye and wing control in either mRNASeq or array data (i.e. genes with fold-change values between minus 2.5-fold and 1.7-fold).

**Figure 5:**
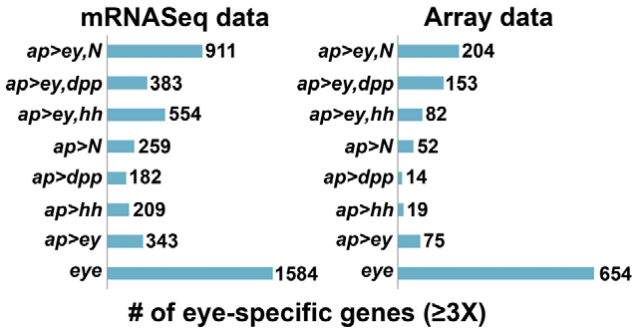
More eye-specific genes are identified as ≥3 upregulated relative to the wing control when Ey+signaling factors are misexpressed compared to when only Ey is misexpressed.

To generate lists of candidate genes whose expression is likely to be controlled by Ey and/or the signaling factors Hh, Dpp or N, and to be important for eye development, we also identified genes ≥3 fold upregulated in both mRNASeq and array libraries generated from *ap>ey*, *ap>hh*, *ap>dpp*, *ap>N*, *ap>ey,hh*, *ap>ey,dpp* and *ap>ey,N* tissue that were also present in the “eye control” gene list (Table S2). A larger subset of the genes in the “eye control” gene list were expressed in wing discs co-expressing *ey* and a signaling pathway (*ey+hh, ey+dpp and ey+N*) compared to when *ey* or the individual signaling pathways were misexpressed alone (Fig 5), again supporting the hypothesis that expression of many eye genes is regulated not by Ey alone, but by Ey in concert with Hh, Dpp or Notch signaling.

### Both eye differentiation and eye fate genes are enriched in the eye control, while the wing control contains mostly wing fate genes

We used DAVID (Database for Annotation, Visualization and Integrated Discovery) [91], [92], [93] to classify candidate genes from each of the lists generated as described above (eye control; *ap>ey; ap>hh; ap>dpp; ap>N; ap>ey,hh; ap>ey,dpp; ap>ey,N*; wing control) (Table S2) into groups according to Gene Ontology (GO) Biological Process and Molecular Function terms (Table S3, S4). In addition, we classified genes from the “No Change” list (Table S5). Because the “No Change” list was too large for the DAVID software to handle, it was necessary to perform the DAVID analysis in three batches (Table S5: No change 1, No change 2, No change 3). We included the “eye control” and “wing control” gene lists as well as the “No Change” gene lists in our DAVID analysis in order to provide information about the transcriptomes of imaginal discs in general and about how the transcriptomes of different imaginal discs vary.

No clusters were identified by DAVID for the *ap>hh*; *ap>dpp* or *ap>N* gene lists, which is not surprising given the low number of genes identified in these lists. In contrast, the “eye control”, *ap>ey*, *ap>ey,hh*, *ap>ey,dpp*, *ap>ey,N*, “wing control” and “No Change” gene lists each had a number of DAVID clusters. A total of 528 DAVID clusters were identified among the three “No Change” batches. Interestingly, there are fewer DAVID clusters for the “wing control” (7) versus “eye control” (55) (Table S3). As discussed in more detail below, this likely reflects the fact that the eye has already begun differentiating at the stage at which the eye-antennal disc tissue was collected, whereas the wing and other structures derived from the wing disc begin differentiating a few hours later.

### Proliferation and epithelial maintenance genes are at similar levels in eye and wing control

Many of the clusters identified in the “No change” lists are linked to metabolic processes that would be expected to occur in all cells and we will not discuss these further. In addition, consistent with the fact that both eye-antennal and wing disc cells proliferate during larval stages, most genes involved in proliferation, growth and maintenance of epithelial cells are found in the “No change” lists. Among these are (1) the small GTPases, including members of the Ras, Rho, Rab, Cdc42, Ran and Arf families, along with the majority of their regulators (one exception is Rab3; see below) (2) cell cycle regulators, components and regulators of the mitotic spindle, and other factors associated with mitosis (3) cell junction assembly/organization; (4) planar polarity and (5) components of the Hippo pathway, which regulates cell-contact-mediated inhibition of proliferation [94], [95], [96], [97], and many others.

### Signaling factor genes are expressed at similar levels in eye and wing control, except for some RTK signaling factors

To our surprise, many signaling factors that have been linked to imaginal disc patterning/cell fate specification (e.g. components of the Hh, Dpp, Notch, Egfr, and Wg signaling pathways, the MAPK/JNK cascades, etc.) are found in the “No change” gene lists. These include the genes encoding the ligands Hh and Dpp, which have very precise patterns of transcription that appear to differ substantially between the wing and the eye-antennal disc (e.g. [45], [98], [99]). At present we do not know the significance of this observation, if any.

One exception is the “RTK signaling” cluster in the eye control DAVID data (cluster #12). The genes in this cluster include *sevenless* (*sev*) and *bride of sevenless* (*boss*), which encode the Sev receptor tyrosine kinase (RTK) and its ligand, which are involved in recruitment of the R7 photoreceptor (reviewed in Freeman, 1997[100]). Another gene in the eye control “RTK signaling” cluster is *roughoid/rhomboid-3* (*ru*), which encodes a serine-type peptidase required for secretion of Egfr ligands that function in photoreceptor recruitment [101]. The presence of the “RTK signaling” cluster in the eye control may therefore reflect the fact that induction of specific eye cell types has already begun in the eye-antennal disc by the stage at which we collected tissue, but will not begin in the wing disc until several hours later.

Interestingly, several genes in the “RTK signaling” cluster encode predicted RTKs about which nothing is known in *Drosophila*, e.g. the predicted insulin-like growth factor receptor encoded by *CG10702*, the predicted fibroblast growth factor receptor encoded by *CG31431*, and the predicted retinoic acid orphan receptor encoded by *Ror*. Future studies are necessary to reveal the potential roles of these genes in eye development.

### Eye and wing control GO clusters contain different sets of tissue-specific transcription factors

In contrast to most of the signaling factors, most of the transcriptional activators associated with tissue specification are differentially expressed in the eye and wing control libraries (Table S2). Accordingly, a “transcription factor activity” cluster is close to the top of the DAVID cluster lists for both “eye control” and “wing control” libraries. For the “wing control” library the “transcription factor activity” cluster had the top enrichment score (7.14), and was comprised of 18 genes that include well-known regulators of wing/dorsal thoracic development (e.g. *ap*, *vg*, *nub*). Similarly, the eye control “transcription factor activity cluster also had one of the top enrichment scores (cluster #2, enrichment score 5.78), and many of the 61 genes in this cluster have previously known roles in eye development (e.g. *eya*, *so*, *dac*, *dan*, *danr*, etc.)

Another large cluster in the wing control library is “wing disc development” (cluster #3, enrichment score 2.14), which contains the GO terms “wing disc morphogenesis” (P Value  = 0.001), “wing disc development” (P Value  = 0.004) and “wing disc pattern formation” (P Value  = 0.03). Other related though less significant clusters are “cell fate commitment/epithelium development” (cluster #5, enrichment score 1.43) and “cell fate commitment/cell morphogenesis” (cluster #6, enrichment score 1.38). Every single gene in these clusters encodes a transcription factor. This is consistent with the fact that the wing disc cells have not yet begun differentiating, but are expressing transcription factors that are important for maintaining the fates of cells that will form adult structures derived from the wing disc.

Consistent with the fact that the eye control library is derived from the eye-antennal disc, which contains the precursors for a number of adult structures, including the eye, the antenna, and most of the rest of the head [89], the DAVID data for the eye control includes multiple clusters containing transcription factors associated with cell fate specification during both eye (orange in Table S3) and antennal development (green in Table S3) (legs and antennae are both limbs and are considered homologous structures).

A number of genes present in the “antennal development” clusters, for instance *dan* and *danr*, have roles in both eye and antennal development [102]. However, the “antennal development” cluster also contains the transcription factor *Lim1*, which has known roles in antennal development and functions to inhibit photoreceptor differentiation [103], [104], [105]. Thus, some of the factors enriched in the eye control library may have roles in development of antennal or other adult head structures but not eye development. Careful analysis of spatial expression patterns and mutant phenotypes will be necessary to draw any firm conclusions about unknown genes enriched in the eye control.

The enrichment of known tissue-specific transcription factors in wing versus eye control respectively confirms that our transcriptomic data is of high quality. However, our DAVID analysis was also useful for identifying potential transcription factors for which no phenotypic data is currently available, and that may have important roles in development of structures derived from the wing or eye-antennal disc. One example for the wing control is the *Homeodomain protein 2.0* (*H2.0*) gene. The vertebrate *H2.0* ortholog, HLX, regulates gene expression in a number of contexts, including during type 1 helper T cell development and in endothelial cells during angiogenesis [106], [107].

For the eye control an example is the *Olig family* (*Oli*) gene, which encodes an Olig family bHLH transcription factor. Interestingly, the two vertebrate Oli orthologs, Bhlhb4 and Bhlhb5, have tantalizing roles in vertebrate retinal development that have not yet been studied in detail [108], [109], [110]. Future analyses of these genes using the powerful genetic techniques available in *Drosophila melanogaster* will greatly enhance our understanding of these genes and their functions in vertebrate eye development and disease.

### Eye and wing control differ in “polysaccharide binding” and “ion binding” genes

Besides the “transcription factor activity” and “wing disc development” clusters mentioned above, which clearly center on cell fate commitment and tissue-specific development, the only other clusters for the wing control are “chitin constituent” (cluster #2, enrichment score 3.96), “polysaccharide binding” (cluster # 4, enrichment score 1.70), and “ion binding” (cluster #7, enrichment score 0.05). All of these clusters have counterparts in the eye control DAVID data (clusters #21, #19 and #44), as well as in the “No Change” DAVID data. For the most part we do not know the significance of these differences in expression of these genes between eye-antennal and wing disc. However, there are a few potentially interesting observations, detailed below.

One of the genes enriched in the eye-control “polysaccharide binding” cluster is *Secreted Wg-interacting molecule* (*Swim*) (*CG3074* in the tables in this paper), which binds to the Wg signaling protein and helps maintain Wg solubility and activity as it diffuses through the extracellular matrix [111]. *Swim* is also upregulated in the *ap>ey,N* library. This data suggests there might be important tissue-specific or stage-specific (e.g. proliferation versus differentiation) differences in diffusion of Wg that have hitherto not been guessed at. Swim is orthologous to vertebrate Tubulo-interstitial Nephitis Antigen (TINag) and its relative TINagL1, which are present and function in the extracellular matrix of a number of organs (c.f. [112]), suggesting that differential regulation of Wnt diffusion could occur in both flies and vertebrates.

Differences in the "ion binding” cluster between the libraries in part reflects the differential expression of tissue-specific zinc finger transcription factors [e.g. *eagle* in the wing control library, and *scratch (scrt)* in the eye control library]. More surprisingly, different cytochrome P450s are found in the “ion binding” clusters in the wing (*Cyp301a1*) versus the eye control (e.g. *Cyp12e1* and *Cyp49a1*) libraries (*Cyp12e1* and *Cyp49a1* are also enriched in the *ap>ey,N* and *ap>ey,dpp* libraries, respectively). Cytochrome P450 s have recently been recognized for roles in developmental biology. For instance, Cyp26 has been found to help shape the retinoic acid gradient important for anterior-posterior patterning in vertebrates [113], [114], [115]. Future work will be necessary to determine what the substrate(s) of the wing- and eye-specific cytochrome P450 s might be, whether they have roles in eye or wing development, and whether the role(s) are conserved e.g. in vertebrate eye development.

### Eye control library is enriched in genes involved in photoreceptor differentiation and function

Unlike the genes enriched in the wing control data, which consist almost entirely of transcription factors involved in wing disc-specific patterning (see above), the eye control genes are much more varied in function. This is clear in the diversity of genes found in individual clusters, as well as in the diversity of clusters themselves. For instance, as described above, the “wing development” cluster in the wing control library includes ***only*** genes encoding transcription factors. In contrast, for the “eye/photoreceptor development” cluster (cluster #4, enrichment score 4.43), only 32/58 of the genes encode transcription factors. Other genes in the “eye/photoreceptor development” cluster encode genes with roles in neuronal differentiation (e.g. axonal pathfinding).

Accordingly, the eye control DAVID data contains clusters associated with neuronal differentiation (red in Table S3). A subset of the genes in the “neuronal differentiation” clusters is listed in Table S6. Some have known roles in photoreceptor differentiation, for instance in photoreceptor axon guidance, including the cell surface protein encoded by *golden goal* (*gogo*) [116], [117], [118], the low density lipoprotein (LDL) receptor repeat-containing protein encoded by *jelly belly* (*jeb*) [119], and the ARID/BRIGHT-family transcription factor *retained/dead ringer* (*retn*) [120], [121]. Also included in these clusters are genes with known roles in neuronal morphogenesis that have not previously been shown to have roles in photoreceptor morphogenesis. One of these is *futsch*, which encodes a microtubule associated protein (MAP) similar to vertebrate MAP1B that is involved in axonogenesis and dendrite morphogenesis as well as synaptic growth at neuromuscular junctions [122], [123], [124], [125]. Future work is necessary to determine whether these genes actually function in axonogenesis or other aspects of photoreceptor morphogenesis.

In addition, a number of DAVID clusters for the eye control library are linked to neuronal/photoreceptor functions (purple in Table S3). The two “detection of light stimulus” clusters (clusters #3 & #8 in eye control; Table S3) contain, for example, several components of the *Drosophila* phototransduction cascade (reviewed in [126]). The enriched genes include *Rh6* (green-light-sensitive rhodopsin expressed in a subset of R8 photoreceptors), *ninaB* (involved in chromophore biosynthesis), and genes involved in terminating the photoresponse, including *rdgC* (encodes rhodopsin phosphatase), and *inaC* (encodes PKC) (Table S8, neuronal function).

A number of genes known to function in synaptic transmission are also found in the eye control library (Table S7, neuronal function). These included genes involved in maintenance of the presynaptic active zone, for instance *bruchpilot* (*brp*) [127], [128], [129], [130], and the small GTPase Rab3 [131]. Finally, a number of genes enriched in the eye control are involved in synaptic vesicle transport and fusion, including the conserved axonal kinesin-3 *unc-104* [132], [133], two genes encoding proteins associated with SNARE function, *neuronal synaptobrevin* (*n-syb*) and *complexin* (*cpx*) [134], and the *couch potato* (*cpo*) gene, which encodes an mRNA-binding protein involved in synaptic transmission and olfactory behavior [135], [136]. The *cpo* gene is a homolog of the vertebrate *RNA binding protein with multiple splicing 1/2 (RBPMS1/2*) genes, which encode members of the RNA recognition motif protein family (RRM). Interestingly, rat *RBPMS* has recently been shown to be a marker for retinal ganglion cells [137].

In summary, our DAVID analysis demonstrates not only that genes important for neural/photoreceptor development are enriched in the eye control, which is expected given that photoreceptors have already begun neuronal development (e.g. projecting axons) at larval stages when the eye-antennal discs were harvested, but also that genes associated more with neuronal function are already expressed in the eye-antennal discs during larval stages.

Since the formation of the rhabdomere (the light-sensing organelle of photoreceptors) only commences halfway through pupal development [138], approximately 2 days after the point at which we collected eye-antennal discs, we did not anticipate enriched expression of phototransduction or synaptic transmission genes in the eye control. However, factors known to regulate *rh6* transcription, for example, including Oc and Sens [74], are expressed in the eye-antennal disc at the larval stages tested [139], [140]. The transcription of *rh6*, and potentially of other neuronal function genes, may therefore be a response to the presence of these factors. An alternative possibility is that the neuronal function genes are expressed in Bolwig's organ, the larval photoreceptor organ, whose nerve extends across the eye-antennal disc and is functional at the time of tissue collection. However, the fact that many of the neuronal function genes are upregulated in the wing disc by *ap>ey*+signaling factors suggests otherwise (see below). The eye control library will therefore be a valuable source for identifying genes not previously known to participate in photoreceptor differentiation and function.

### Eye control contains genes for gland, mesoderm and muscle development, immune response

There are a few surprising clusters with fairly strong enrichment scores in the eye control library, including “gland development” (cluster #6), “immune response” (cluster #11), “mesoderm development” (cluster #18), “programmed cell death” (cluster #27), “heart development” (cluster #30) and “muscle development” (cluster #36). In part this reflects the fact that many factors have pleiotropic roles in development. A good example of this is *eya*, which in addition to eye development has a role in development of somatic and ventral mesoderm, including the precursors of the salivary gland [141], [142].

It is also possible that other tissues got incorporated into the eye-antennal disc tissue preparations. For instance, the three “Halloween” class of genes, *disembodied* (*dib*), *shadow* (*sad*) and *spookier* (*spok*), are expressed in the ring glands but not in epidermal structures such as the eye-antennal discs [143], [144], [145], [146], [147], [148]. All three are expressed in the prothoracic gland cells of the ring glands but not in epidermal structures such as the eye-antennal imaginal disc. Since the ring glands lie between the pair of eye-antennal discs in the larval head, it is possible that parts of the ring gland were included with the eye-antennal discs into our tissue preparations.

Nevertheless, although a complete analysis of the genes in these unexpected clusters is beyond the scope of the present paper, there are likely to be some interesting genes in these clusters whose functions in eye development are worth pursuing.

### The *ap>ey*+signaling factor libraries are enriched in genes involved in eye development and neural/photoreceptor differentiation and function

As expected based on our analysis above (Fig. 4; Table S1), many of the well-known genes encoding transcription factors involved in eye/photoreceptor specification are present in the “eye/photoreceptor development” clusters near the top of the *ap>ey*+signaling factor DAVID lists. In fact, with two exceptions (*pros* and *toy*), all of the 13 eye/photoreceptor specification genes present in the eye control “eye/photoreceptor development” cluster are also present in the *ap>ey,dpp* “eye/photoreceptor development” clusters (Table S8) (as mentioned above, *toy* functions upstream of *ey* and isn't expected to be present in *ap>ey*+signaling factor libraries. A number of genes in the eye control “RTK signaling” cluster are present in the *ap>ey*+signaling factor “eye/photoreceptor development” clusters, including *sev* (*ap>ey*, *ap>ey,hh*, *ap>ey,dpp*, *ap>ey,N*), *ru* (*ap>ey*, *ap>ey,hh*, *ap>ey,dpp*), *boss* (*ap>ey,dpp*) and *Ror* (*ap>ey,dpp*). Thus, Ey and especially Ey+Dpp are capable of activating high levels of expression of a large percentage of the eye/photoreceptor transcription factor genes and RTK signaling genes in the wing disc.

As in the eye control library, the DAVID software identifies clusters associated with neuronal differentiation in all of the *ap>ey*+signaling factor libraries (red in Table S3), as well as clusters associated with neuronal function in the *ap>ey,hh* and *ap>ey,dpp* libraries (purple in Table S3). These clusters contain several of the “neuronal differentiation” and “neuronal function” genes present in the eye control (Tables S6, S7), suggesting that Ey or Ey+signaling factors activate their expression directly or indirectly. Of the genes mentioned above in the eye control DAVID analysis, these include *gogo* (*ey+dpp*), *retn* (*ey+dpp*) and *futsch* (*ey+dpp*) for “neuronal differentiation” and *ninaB* (*ey+dpp*), *inaC* (*ey*, *ey+hh*, *ey+dpp*), *n-syb* (*ey+dpp*) *cpx* (*ey+N*) and *cpo* (*ey+dpp*) for neuronal function. Interestingly, the *ey+dpp* library appears to be more enriched compared with the other libraries in genes with known or suspected roles in neuronal differentiation or function compared with the other *ap>ey*+signaling factor libraries.

In summary, the transcriptome downstream of Ey+Hh,Dpp,N includes not only genes encoding transcription factors and signaling molecules important for cell fate specification, but also factors involved in neuronal/photoreceptor differentiation and function. In addition, the fact that Ey+Hh,Dpp,N are capable of activating expression of neuronal/photoreceptor differentiation and function genes in the wing disc indicates that these genes are expressed as part of normal eye development, as opposed to expression in the Bolwig's organ.

### The eye control, *ap>ey+dpp* and *ap>ey*+N libraries are enriched in peptidases

One unexpected group to emerge from our DAVID analysis was the “peptidases”, for which there are clusters in the eye control library (cluster #33), as well as the *ey+dpp* (cluster #14) and *ey+N* (cluster #3) libraries (Table S3, S4). Many of the genes in the cluster are predicted genes with sequence homologies to known peptidases, but about which nothing further is known in *Drosophila*. The enriched peptidases include a number of predicted serine peptidases, several metallopeptidases, and a single predicted cysteine endopeptidase encoded by the *CG3074/Swim* gene (described above) (Table S9); Swim is probably not catalytically active [111].

Several of the metallopeptidases enriched in the eye control have clinically important human orthologs. For instance, *Ance-5* encodes an angiotensin-converting enzyme ortholog, *CG14516* encodes an aminopeptidase N/CD13 ortholog, *CG4408* encodes a pancreatic carboxypeptidase A1 relative, *Mmp2* encodes a matrix metallopeptidase and *Tace* encodes an ortholog of ADAM17/tumor necrosis factor-α-converting enzyme (TACE). With the exception of *Mmp2* [149], [150], there is no phenotypic data available in *Drosophila* for any of these genes.

In addition to the peptidases themselves, a few peptidase regulators are enriched in the eye control library. One is *7B2*, which encodes a *Drosophila* ortholog of 7B2. Vertebrate 7B2 is a chaperone for the prohormone convertase 2 (PC2) enzyme; interestingly, both PC2 and 7B2 are upregulated by vertebrate Pax6 in the pancreas [151]. The other peptidase regulator enriched in the eye control library is the *Serine protease inhibitor 1* (*Spn1*) gene, which encodes a serpin that can inhibit trypsin *in vitro* and plays a role in immune response to fungal infection in *Drosophila* [152]. Neither 7B2 nor Spn1 has a known role in eye development.

A number of peptidase genes enriched in the eye control library are also enriched in the *ap>ey*, *ap>ey+hh*, *ep>ey+dpp* and *ap>ey+N* libraries. In particular, 10 out of 26 peptidase genes plus the serpin *Spn1* gene are enriched in *ap>ey,N*, by far the most of any of the other *ap>ey*+signaling factor libraries. Interestingly, the list of peptidase-encoding genes in the *ap>ey,dpp* and *ap>ey,N* “peptidase” clusters is mutually exclusive. These results suggest that peptidases have important roles during *Drosophila* eye development, and that they are part of the transcriptome downstream of Ey+Hh,Dpp,N.

### The *CG4721* gene is strongly upregulated by Ey+N and has a role in eye development

To determine whether any of the genes identified through mRNASeq/microarray have roles in eye development, we obtained *UAS-RNAi* strains for candidate genes from the Vienna *Drosophila* RNAi Center (VDRC). We crossed these strains to a number of *Gal4* strains (*ey-Gal4*, *dan-Gal4*, *mirr-Gal4*, *GMR-Gal4*, *np2631-Gal*4, *sev-Gal4*, etc.) to drive expression in various temporal and spatial patterns during eye development. This approach uncovered a strong phenotype for the *RNAi* strain targeting the *CG4721* gene.

**Figure 6:**
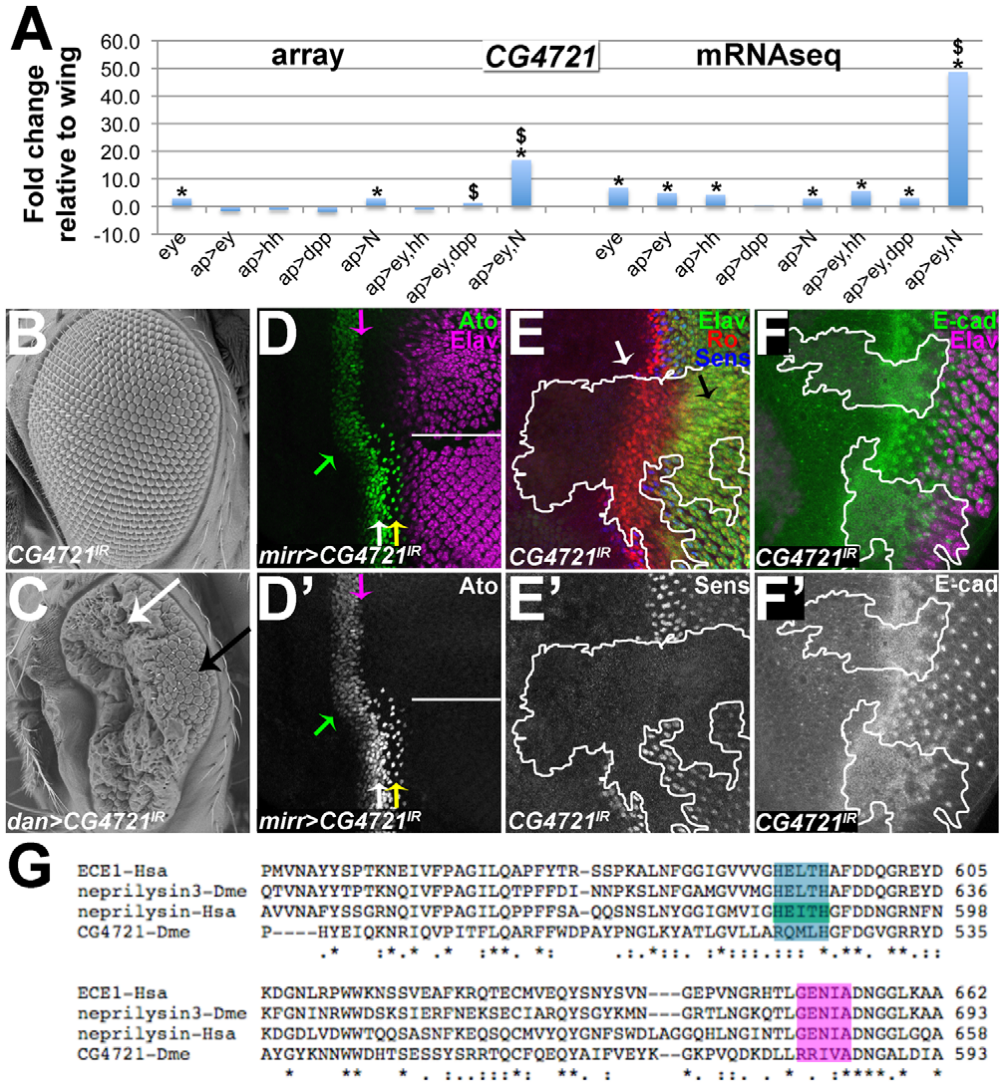
*CG4721* encodes a neprilysin that is required for eye development. (A) Graph showing fold-changes in *CG4721* levels in the indicated genotypes. “*”indicates significant upregulation; “$” indicates signficant upregulation in *ap>ey*+*N* factor versus both *ap>ey* and *ap>N*. (B,C) Scanning electron micrographs of adult eyes of the indicated genotypes. (D) Projection of confocal sections of *mirr>CG4721^IR^* eye-antennal discs stained for anti-Ato and anti-Elav. White line divides dorsal (top) from ventral (bottom). (E,F) Projection of confocal sections of eye-antennal discs containing clones expressing *CG4721^IR^* (outline of clones marked by white lines). (E) *CG4721^IR^* tissue fails to develop R8s (marked by Sens expression). Instead, all *CG4721^IR^* cells behind the furrow express Ro and develop as R2/R5 photoreceptors (marked by Ro and Elav). (F) E-cad, which marks the furrow in wild-type tissue, is expressed in a broader domain in *CG4721^IR^* tissue compared to surrounding wild-type tissue. (G) Alignment of the catalytic domain of the predicted CG4721 protein sequence with the catalytic domain of other neprilysins from *Drosophila melanogaster* and *Homo sapiens*. Colored boxes indicate residues critical for catalytic activity.

Microarray and RNASeq data reveal that *CG4721* is expressed at higher levels in control eye-antennal discs compared to control wing discs (Fig 6A). Whereas *CG4721* is slightly upregulated in response to N misexpression in the wing disc, it is very strongly upregulated in response to Ey+N misexpression in the wing disc (∼18-fold in the array data and ∼48-fold in the mRNASeq data). Thus, *CG4721* appears to be a target of Ey and N during eye development.

The *dan-Gal4* strain drives expression in and behind the morphogenetic furrow [153]. Whereas eyes from parent *UAS-CG4721^IR^* and *dan-Gal4* flies appear normal (Fig 6B and not shown), eyes from *dan>CG4721^IR^* flies are smaller and are misshapen (Fig 6C). They consist of a patch of 10-20 relatively normal-appearing ommatidia at the posterior of the eye portion of the eye-antennal disc near the point at which the morphogenetic furrow initiates (Fig 6C, black arrow) with a highly distorted retinal structure anteriorly (Fig 6C, white arrow). Other Gal4 strains that drive expression in and around the furrow (e.g. *np2631-Gal4*–[154]) and in the dorsal half of the eye (*mirr-Gal4*– [155]) also gave strong eye phenotypes when crossed to *UAS-CG4721^IR^* (not shown).

**Figure 7:**
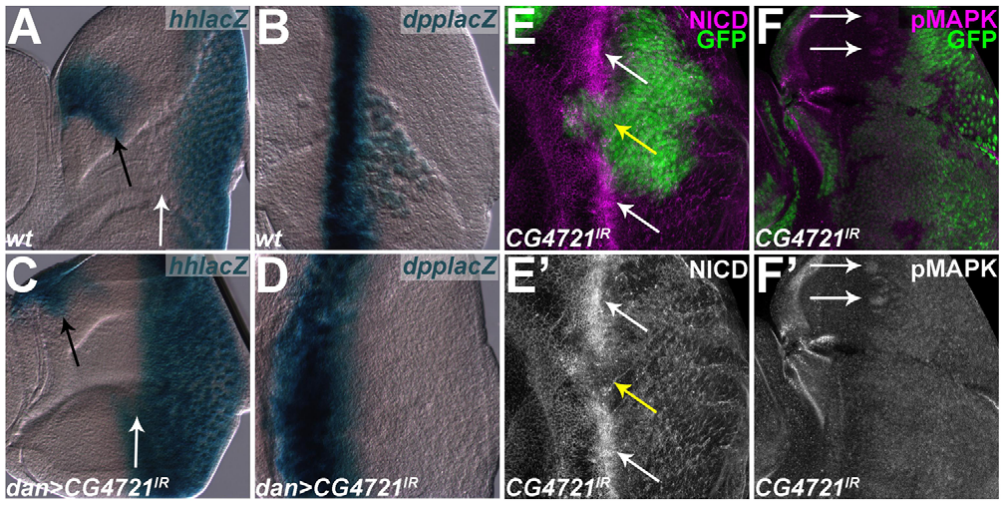
Eye-antennal discs from otherwise wild-type *hh-lacZ* (A) or *dpp-lacZ* (B) larvae. (A) *hh-lacZ* is expressed in photoreceptors behind the furrow (white arrow) and in the ocellar precursors (black arrow). (B) *dpp-lacZ* is expressed in the furrow. (C) *dan>CG4721^IR^/hh-lacZ* eye-antennal disc. *hh-lacZ* expression in ocelli is normal (black arrow), but expression in eye field is advanced relative to wild type (A). (D) *dan>CG4721^IR^/dpp-lacZ* eye-antennal disc. *dpp-lacZ* is expressed in a broader domain compared to wild type (B). (E,F) Projection of confocal sections of eye-antennal discs containing clones expressing *CG4721^IR^* (clones marked by GFP). (E) NICD is enriched in the furrow in wild-type tissue (white arrows), but is not enriched in the furrow in *CG4721^IR^* tissue (yellow arrow). (F) pMAPK is enriched in intermediate groups in wild-type tissue (white arrows), but is enriched in all cells in *CG4721^IR^* clones.

To probe the mechanisms behind these dramatic effects on eye development, we stained larval *mirr>CG4721^IR^* eye-antennal discs with antibodies against the bHLH factor Atonal (Ato), which is the proneural transcription factor for eye development and is required for eye development [156], [157]. In the wild-type ventral half of *mirr>CG4721^IR^* discs, Ato is first expressed at low levels ahead of the furrow in all cells (Fig 7D, white arrow). As the furrow propagates, Ato is upregulated in a process called proneural enhancement, and becomes limited first to “intermediate groups” of ∼15 cells, then to “R8 equivalence groups” of 2-3 cells and finally to single R8 cells (Fig 7D, yellow arrow), which are the founding cells of each developing ommatidium [156], [157], [158], [159], [160], [161]. In contrast, in the mutant dorsal half of *mirr>CG4721^IR^* eye-antennal discs Ato expression initiates further ahead of the furrow compared to wild type (Fig 7D, green arrow). In addition, proneural enhancement of Ato in intermediate groups does not occur in the mutant dorsal half of *mirr>CG4721^IR^* eye-antennal discs, and very few R8 cells expressing Ato emerge from the furrow (Fig 7D, magenta arrow). These results suggest that the *CG4721* gene has a role regulating Ato expression.

Transcription of the *ato* gene is under the control of two distinct *ato* regulatory elements: the 3′enhancer and the 5′enhancer: the 3′ enhancer controls the initial upregulation of *ato* in all cells anterior to the furrow; the 5′ enhancer is required for *ato* expression in intermediate groups and in single R8 precursors [25], [162]. We used *3*′ *ato-GFP* and *5*′ *ato-lacZ* reporter genes to further analyze the effects of CG4721 reduction on *ato* expression.

As with Ato protein expression, in *dan>CG4721^IR^/3*′ *ato-GFP* eye-antennal discs GFP is expressed further anterior to the furrow compared to wild type (white bars in Fig S5A,B). In addition to expression in the ocellar precursors (Fig S5C,D, white arrow), *5*′ *ato-lacZ* is detected in all R8s posterior to the MF in the eye portion of wild type eye-antennal discs, likely due to perdurance of β-Galactosidase (Fig S5C). In contrast, although the *5*′*ato-lacZ* reporter gene is expressed in ocellar precursors in *dan>CG4721^IR^ /5*′*ato-lacZ* eye-antennal discs (Fig S5D, white arrow), only a few weak spots of *lacZ* activity were observed in the eye field. These results are consistent with our earlier observations of Ato protein expression in *CG4721^IR^* eye-antennal discs. This indicates that CG4721 regulates *ato* expression at the transcriptional level, and suggest that CG4721 affects input into both the 3′ and 5′ *ato* enhancers.

### Non-R8 photoreceptors are recruited in *CG4721* RNAi eye-antennal discs

Expression of Ato in the R8 precursor is required for R8 specification [156], which in turn is required for recruitment of all of the other photoreceptors in the following order: 2/5, 3/4, 1/6 and 7 [163]. Given that *CG4721* is required for Ato expression in R8 precursors (Fig. 6D), we predicted that *CG4721^IR^* eye tissue posterior to the MF would lack R8s and all other types of photoreceptors. To test this idea we stained eye-antennal discs containing GFP-marked *CG4721^IR^* clones with anti-Senseless (Sens), which marks R8s and is an Ato target [139], [164], and with anti-Elav, which is a pan-neural protein that marks photoreceptors [165]. As expected, Sens is not expressed in *CG4721^IR^* clones (Fig 6E; clone marked by white line). These results suggest that CG4721 is required for R8 cell differentiation.

Interestingly, as indicated by anti-Elav staining, numerous neurons are recruited in *CG4721^IR^* tissue even in the absence of R8 specification (Fig. 6D,E). To further explore the *CG4721^IR^* phenotype and to determine the identity of the neurons present in *CG4721^IR^* tissue, we stained discs containing *CG4721^IR^* clones with antibodies against Rough (Ro). Ro is expressed in a mutually exclusive pattern with Ato and Sens, with Ro expression initiating in cells that lie between Ato intermediate groups as they emerge from the initial broad swath of Ato expression. Later, Ro is a marker for photoreceptors R2/R5 and R3/R4, and is capable of converting R8 precursors to an R2/R5 fate [164]. Consistent with its expression pattern, Ro is a negative regulator of *ato* expression and is capable of repressing Ato expression when expressed ectopically. Conversely, Sens is a negative regulator of Ro in the R8 precursor, allowing for proper development of R8 ([160], [164], [166] Fig 6E).

In *CG4721^IR^* clones Ro expression initiates a few cell rows more anteriorly than in surrounding wild-type tissue (Fig 6E, white arrow). In addition, Ro appears to be expressed in every cell in *CG4721^IR^* clones: all of the Elav-expressing cells also express Ro (Fig 6E, black arrow). These results suggest that Ro expression initiates too early in *CG4721^IR^* tissue, possibly resulting in Ato repression in intermediate groups. In the absence of Ato, Sens expression and R8 differentiation fail. Instead, the high levels of Ro expression in all cells lead to their conversion to an R2/R5 fate. This suggests that CG4721 is responsible for activating Ato and repressing Ro.

### CG4721 is required for normal furrow morphology

Premature Ato and Ro expression in *mirr>CG4721^IR^* discs and in *CG4721^IR^* clones suggested that the furrow itself might accelerate compared to wild type (Fig 6D,E). We therefore examined furrow morphology by staining eye-antennal discs containing *CG4721^IR^* clones with an antibody against *Drosophila* E-cadherin (*D*E-cad). During wild-type eye development *D*E-cad is upregulated in furrow cells, which undergo an apical constriction. A subset of cells that emerge from the furrow maintain high levels of *D*E-cad expression and form clusters that are the precursors of ommatidia. Cells in between the clusters down-regulate *D*E-cad to pre-furrow levels [167]. In *CG4721^IR^* clones that span the furrow (Fig 6F), *D*E-cad appears to be up-regulated earlier compared to surrounding wild-type tissue, which is consistent with our observations of Ato and Ro. In addition, high levels of *D*E-cad are maintained in all cells well posterior of where it is ordinarily down-regulated between clusters.

### CG4721 encodes a neprilysin

*CG4721* encodes a protein of 686 residues that is predicted to be a member of the neprilysin (M13) family zinc-metalloendopeptidases [168]. M13 peptidases are type II transmembrane proteins and are also known as “Glu-zincins”. They have two consensus sequences that are important for coordinating the zinc ion that is essential for catalysis [168], [169], [170]. The first consensus sequence is a typical HExxH sequence (Fig 6G, turquoise box), with the His residues forming two of the three zinc ligands, and the Glu residue functioning in catalysis. The second consensus sequence is ExxA/GD (Fig 6G, magenta box), with the Glu residue serving as the third zinc ligand.

Based on phylogenetic analysis [168], *CG4721* falls into a *Drosophila*-specific clade of M13 peptidases, with no direct human orthologs. Although the sequences of members of this clade indicate that they fall into the neprilysin family of proteins, some members including CG4721 lack key catalytic residues (Fig 6G), and probably have non-catalytic functions. At least one other member of the M13 family has a non-catalytic function: mammalian PHEX has been shown to regulate the activity of the extracellular matrix protein MEPE, which is an inhibitor of phosphate uptake and mineralization, via a non-catalytic direct interaction [171], [172]. It is possible that the *CG4721* protein regulates *Drosophila* eye development via a similar mechanism.

### CG4721 regulates Hh, Dpp and/or EGFR signaling pathways

Based on the fact that neprilysins are type II transmembrane proteins with the bulk of the protein sequence in the extracellular domain, as well as the fact that neprilysins are known for their roles in inactivating signaling peptides [168], we predicted that the regulation of *ato* by CG4721 would be indirect through one of the key eye signaling pathways (Hh, Dpp, Egfr or Notch).

All four of these signaling pathways are involved in an autoregulatory loop that promotes progression of the furrow (reviewed in Roignant and Treisman, 2009 [173]). To summarize, in response to Egfr signaling, Hh is expressed in photoreceptors behind the furrow, and activates *dpp* expression in the furrow. Hh and downstream signals act redundantly to turn off repressors of Ato, allowing for Ato expression to initiate at low levels. In addition, Hh and Dpp are redundantly required for expression of the Notch ligand Delta in the furrow [174]. Notch signaling, activated by Delta, is required for upregulation of Ato levels during proneural enhancement [30], [159], [175]. Ato becomes restricted to individual R8 precursors via lateral inhibition, and leads to secretion of the Egfr ligand Spitz from R8. Spitz activates Egfr signaling in surrounding cells, recruiting them as photoreceptors and activating Hh, thus completing the autoregulatory loop.

To examine the effects of *CG4721^IR^* on Hh and Dpp expression, we performed a *lacZ* reporter assay with *hh-lacZ* and *dpp-lacZ* in *CG4721^IR^* eye-antennal discs. In a wild-type eye-antennal disc, *hh-lacZ* is expressed in developing photoreceptors posterior to the MF (Fig. 7A), while *dpp-lacZ* is expressed in a thin strip of cells that lie in the MF (Fig. 7B) [98], [99]. However, in *dan>CG4721^IR^* eye-antennal discs, *hh-lacZ* is expressed more anteriorly than in wild-type discs (Fig. 7C) and *dpp-lacZ* is expressed in a broader band (Fig. 7D) compared to wild type. These data are consistent with the apparent acceleration and broadening of the furrow observed in *CG4721^IR^* tissue based on Ato, Ro and *D*E-cad expression (Fig 6).

In *CG4721^IR^* eye-antennal discs, Ato expression does not appear to undergo proneural enhancement, which requires Notch signaling [30], [159], [175]. To test the effects of *CG4721^IR^* on Notch pathway activity, we stained discs containing GFP-marked *CG4721^IR^* clones with antibodies against the intracellular component of the Notch receptor (NICD). NICD serves as an indicator of N activity given that when the N receptor is activated upon binding to its ligand (Delta or Serrate), the intracellular component is cleaved and transported to the nucleus where it co-regulates expression of target genes (reviewed in Fortini, 2009 [176]). In the eye portion of wild-type eye-antennal discs, NICD is enriched in the cytoplasmic membrane of the cells in the morphogenetic furrow (Fig 7E, white arrows). However, in *CG4721IR* clones NICD was down-regulated (Fig. 7E, yellow arrow), suggesting that the Notch signaling pathway requires CG4721 for its activity.

As shown in Fig 6E, *CG4721^IR^* eye tissue lacking R8s is still able to recruit photoreceptors. Moreover, *D*E-cad is maintained in all cells behind the furrow in *CG4721^IR^* tissue (Fig. 6F). Dominguez *et al.* (1998) [177] have demonstrated that ectopic activation of Egfr signaling induces formation of photoreceptors even in an *ato* loss-of-function background. Furthermore, Egfr has a known role in maintaining high *D*E-cad levels in ommatidial preclusters posterior to the furrow [167]. This led us to suspect that Egfr activity might be elevated in the absence of CG4721.

Accordingly, we stained discs containing *CG4721^IR^* clones for phosphorylated MAPK (pMAPK). Unlike in wild-type eye tissue where pMAPK is detected only in intermediate cell clusters emerging from the MF (white arrows in Fig. 7F), pMAPK was found to be up-regulated in all cells in *CG4721^IR^* clones that span the MF (Fig. 7F). This suggests that the recruitment of photoreceptors and maintenance of *D*E-cad in *CG4721^IR^* tissues is likely due to Egfr upregulation.

Taking all of this data together, and considering that furrow progression is controlled by an autoregulatory loop involving Hh, Dpp, N and Egfr signaling [173], a couple of things stand out. One is that, although high Egfr signaling levels are known to depend on Ato expression during normal eye development [178], [179], in *CG4721^IR^* tissue Egfr signaling levels are high in the absence of Ato. This suggests that during wild-type eye development *CG4721* is involved (directly or indirectly) in repressing Egfr signaling until Ato expression is activated.

The fact that Pointed, the transcription factor activated by the Egfr signaling pathway, is known to directly activate Hh expression [180] is consistent with the broader expression domains of *hh-lacZ* and of *dpp-lacZ* (since *dpp* is a target of Hh signaling) in *CG4721^IR^* tissue.

Why then, does proneural enhancement and subsequent expression of Ato fail? After all, proneural enhancement and subsequent steps in eye development ultimately depend on Hh and Dpp. For instance, Hh and Dpp signaling are required redundantly for Delta expression [174], which then signals through N to allow for Ato upregulation during proneural enhancement [30], [159], [175]. Interestingly, in spite of the fact that *hh* and *dpp* are clearly expressed in *CG4721^IR^* tissue (Fig 7C,D), Notch activity levels are reduced (Fig 7E). Thus, *CG4721* has a role in N pathway activation downstream of Hh and Dpp, and the loss of N activity occurring in *CG4721^IR^* tissue may prevent Ato proneural enhancement, and Ato's subsequent maintenance via autoregulation.

The high levels of the Ato repressor Ro observed in *CG4721^IR^* tissue suggest an alternative though not necessarily mutually exclusive possibility for why Ato expression is never upregulated and is instead lost. Both Hh and Egfr are required for Ro expression, and Hh can activate Ro expression ectopically [177], [181]. It is possible that the high levels of Egfr observed in *CG4721^IR^* tissue lead to Ro activation, directly and/or through Hh signaling. Ro would then repress Ato expression, but influence the identity of the photoreceptors recruited through Egfr signaling, such that they all take on an R2/R5 fate (Fig 6E). Future experiments will be necessary to distinguish between these possibilities and to identify CG4721 targets.

## Discussion

Patterned specification is a term that can be used to describe the interplay between tissue-specific transcription factors and signaling pathways that is necessary for development of tissues and organs containing multiple cell types. For instance, during *Drosophila* eye development the Pax6 homolog Ey defines the organ type (“eye”), while signaling pathways including Hh, Dpp and N contribute necessary spatial and temporal information by mediating cell-cell communication.

At some level, transcription factors such as Ey cooperate with signaling-pathway-specific transcription factors to co-regulate appropriate patterns of transcription of genes responsible for organ development [1], [2], [3]. A number of previous studies have used high-throughput methods to identify Ey/Pax6 targets in the developing *Drosophila* eye [24], [182] and in a number of contexts in vertebrates [183], [184], [185], [186], [187], [188]. However, none of these studies has examined the effect of signaling factors in the development of structures that require Pax6 function. An approach that combines analyses of Ey/Pax6 and signaling pathways will (1) identify more of the genes important for eye development, thereby clarifying the nature of the eye transcriptome; (2) provide clues as to the mechanisms by which the signaling and specification factors combine to regulate transcription during development.

We have harnessed the power of shotgun, clonal mRNA sequencing from Illumina to identify genes whose transcription is regulated by Ey+Hh, Ey+Dpp or Ey+N. As discussed below, several analyses suggest that this approach provides a more complete snapshot of eye development than would a focus on Ey alone. Firstly, unsupervised principal component and two-way hirerarchical clustering analyses demonstrate that the *ap>ey+*signaling factor transcriptomes are more similar to the eye control transcriptome than the *ap>ey* transcriptome is.

Moreover, a greater number and diversity of eye-specific genes were ≥3-fold upregulated in the *ap>ey+*signaling factor transcriptomes compared to the *ap>ey* transcriptome. In terms of numbers, of the 212 eye-specific genes ≥3-fold upregulated in *ap>ey,hh*, *ap>ey,dpp* or *ap>ey,N* wing discs, only 42 were also found in the list of eye-specific gene ≥3-fold upregulated by *ap>ey*, leaving 170 that potentially would not have been identified if we had focused solely on Ey. In addition, the DAVID analysis demonstrates the greater diversity of genes in the *ap>ey*+signaling factor transcriptomes, with a greater enrichment of both transcription factors and cellular factors involved in regulating neuronal shape, structure and function present in *ap>ey*+signaling factor libraries (particularly the *ap>ey,dpp* library) compared to the *ap>ey* library.

The genes found to be unique to the *ap>ey*+signaling factor libraries also include 92 genes denoted only by a CG number, which is a wealth of uncharacterized genes with potential functions in eye development. A number of these encode putative peptidases, which based on DAVID analysis are enriched in the eye control versus the wing control, and are also found in two of the *ap>ey*+signaling factor libraries (*ap>ey,dpp* and *ap>ey,N*) but not in the *ap>ey* library. One of these putative peptidases is CG4721, which was not identified as significantly upregulated by Ey expression either in previous microarray analyses [24], [182] or in our *ap>ey* wing discs, but which we found in the *ap>ey,N* library.

As described above, reduction of *CG4721* function results in an extremely interesting phenotype affecting initiation of the proneural gene *ato*. Expression of a number of factors that regulate *ato* expression, including the transcription factor Ro and the signaling ligands *hh* and *dpp* are also affected, as is signaling activity for the Notch and Egfr pathways (Figs 6,7). Regulating *ato* expression is critical because it leads to specification of single, spatially patterned R8 precursors, which subsequently leads to recruitment of other cell types. A similar process occurs in the vertebrate retina, which involves the Ato homolog Ath5 [189], though much less is known about Ath5 regulation.

In summary, our analysis of the transcriptomes downstream of Ey, Ey+Hh, Ey+Dpp and Ey+Notch will be a valuable resource for researchers trying to understand the network of genes that promote eye development in both *Drosophila* and vertebrates. Future efforts will be aimed towards identifying direct transcriptional targets of Ey as well as the Hh, Dpp and Notch signaling pathways, using both *in silico* and experimental approaches, and analyzing the biological and molecular functions of the genes identified.

### The *ap>ey,hh* transcriptome is closer to the eye control than *ap>ey,dpp* or *ap>ey,N*

It is interesting to compare the different signaling pathways with respect to the transcriptomes obtained as a result of co-misexpression with Ey. For instance, most of the previously known eye genes examined responded most strongly to the Ey+Dpp combination, with Ey+Hh and Ey+N often having less of an effect than Ey alone (Fig 4B–D; Table S1). Furthermore, Ey+Dpp and especially Ey+N had a greater number of eye-specific genes expressed at ≥3-fold versus the wing control compared to Ey+Hh (Fig 5; Table S2).

Finally, for the DAVID analysis Ey+Dpp had the greatest diversity of GO clusters compared to Ey+Hh and Ey+N, and was most similar to the eye control in terms of types of GO clusters obtained and the number and identity of genes in the clusters (Tables S3, S4, S6, S7, S8, and S9). For instance, comparing Ey+Hh, Ey+Dpp and Ey+N for the main categories of clusters discussed for the eye control library: eye-specific genes, neuronal differentiation genes, neuronal function genes and peptidase genes, Ey+Dpp had the most genes in every category except peptidases. Ey+N had most of the peptidase genes, including 3 in the neprilysin family (one of which is *CG4721*). It is currently not clear why peptidases in general should be upregulated by the Ey+N combination (see below for discussion of *CG4721*). By these criteria, one might be tempted to speculate that Ey+Dpp or Ey+N is more effective at recreating eye development than Ey+Hh.

In contrast, much experimental data indicates that Hh signaling acts upstream to regulate activity of the Dpp and Notch pathways during eye development, e.g. through direct transcriptional control of *dpp*, although there is some redundancy among the pathways with respect to regulating eye gene expression [31], [32], [173], [190]. Moreover, by our principal components analysis and two-way hierarchical clustering, the *ap>ey,hh* transcriptome is more similar to the eye control compared to *ap>ey,dpp* and *ap>ey,N*, at least for the mRNAseq data. These observations suggest that Hh is at the top of a signaling hierarchy for the eye.

How can we resolve these apparently conflicting results? It could be argued that, since the eye-antennal disc contains more than just eye precursors, it is not the best tissue to use for comparison in terms of an idealized “eye transcriptome”. Alternatively, it is possible that the differences in transcriptomes between *ap>ey*, *ap>ey,hh*, *ap>ey,dpp* and *ap>ey,N* is due to differences in transgene expression levels (e.g. *UAS-hh* vs. *UAS-dpp*), resulting in fewer genes that would consequently fall into fewer GO categories. The fairly high threshold expression-fold level (≥3-fold) used to determine which genes were included in the DAVID analysis could have augmented these differences. In contrast, the principal components analysis included all the data.

Along these lines, it is possible that Ey+Hh balances both activating and inhibiting inputs into eye development, for a more comprehensive regulation of eye gene expression, for instance in terms of expression levels. Thus, the list of genes that are so strongly activated by misexpression of Ey+Dpp and Ey+N may reflect the absence of the inhibitory factors that would be triggered by Ey+Hh signaling, resulting in expression levels that are more proportional to those found during normal eye development. Indeed, Ey+Dpp and Ey+N activate expression of a number of genes at levels that are many-fold higher than the fold-difference between the eye and wing control, including *ey* and *CG4721*. Future work will be necessary to distinguish among these possibilities, and will undoubtedly reveal interesting differences in Hh, Dpp and Notch targets in eye development.

### The eye-antennal disc transcriptome is enriched in genes with roles in neural/photoreceptor differentiation and function

The imaginal discs of holometabolous insects including *Drosophila melanogaster* are epithelial structures set-aside during embryonic stages as the precursors of external adult structures such as the antennae, eyes, legs, wings and cuticular structures of the head and thorax. During larval development, the 10–50 imaginal disc cells (depending on the disc) proliferate to generate 10,000–50,000 cells, while maintaining the identity of the segment from which they are derived (e.g. dorsal part of the 2nd thoracic segment, in the case of the wing disc).

The *Drosophila* imaginal discs have been used to study everything from the mechanisms of asymmetric cell division [191] to the mechanisms of tissue repair and regeneration [192], [193], [194]. However, although a number of studies have generated transcriptome data (generally microarray data) from *Drosophila* imaginal discs (eg. [24], [182]), we are unaware of studies that have analyzed the similarities and differences between imaginal disc transcriptomes. This information will be useful for interpretation of data obtained in numerous studies, as well as for identifying novel genes for further study.

As expected, based on a DAVID analysis of the eye-antennal and wing disc transcriptomes, the vast majority of genes are expressed at similar levels between eye-antennal and wing discs (“No change” in Tables S2,S5). In addition to genes involved in general metabolism, other genes in the “No change” list encode proteins important for regulating the cell cycle and maintaining epithelial structure. Given that both the eye-antennal and wing discs are both epithelial structures with a large proportion of cells undergoing continuous proliferation, the fact that these genes are expressed at similar levels in both eye and wing control libraries was not a surprise.

In contrast, the proportion of genes in the eye and wing control libraries encoding sequence-specific DNA-binding transcription factors was quite high. This makes sense given the importance of maintaining disc identity while proliferation is occurring. Accordingly, many of the transcription factor genes that are differentially expressed between the eye and wing control have important roles in tissue specificity: eg. *ey*, *eya*, *so*, *dac* for the eye control; *ap* and *vg* for the wing control.

The enrichment in transcription factors is particularly striking in the case of the wing disc. At the time of tissue dissection, approximately one-third of the eye-antennal disc cells have already begun to differentiate into photoreceptors, and have begun extending axons and undergoing other forms of morphogenesis. In contrast, cells in the wing disc are still proliferating and undergoing patterning, and will not begin differentiating for a few more hours. Consequently, there were few clusters of enriched genes obtained by DAVID analysis for the wing control: only 7 (Table S3), and most contained almost exclusively transcription factor genes, particularly those such as *ap* and *vg* that are important for specification and maintenance of wing cell fate.

For the eye control library, on the other hand, we obtained 55 DAVID clusters (Table S3). Similar to the wing disc, many of these clusters are also associated with cell fate specification/maintenance of cell fate appropriate to the different tissues derived from the eye-antennal disc (eye, antenna, etc.). In addition, a total of 8 clusters were related in some way to neuronal differentiation, and 7 clusters were related to neuronal function. In part, the number and type of clusters undoubtedly reflects the fact that photoreceptor neurons have already begun differentiation at the time the discs were dissected.

However, we were not expecting genes with roles in neuronal function to be expressed in the eye-antennal disc, since photoreceptors are not functional until considerably later. Interestingly, some of the genes involved in neuronal function are activated in the wing disc by *ap>ey*, *ap>ey,hh*, *ap>ey,dpp* and *ap>ey,N*. The DAVID data for the *ap>ey,dpp* wing discs in particular contains clusters that correspond to 4 of the 8 neuron-related clusters. Future work will be needed to understand the network leading from expression of Ey and the signaling molecules to the expression of genes important for neuronal function.

Finally, one unexpectedly interesting cluster found in the eye control (#33), *ap>ey,dpp* (#14) and *ap>ey,N* (#3) libraries but not in the wing control library is the peptidase cluster. In part this caught our attention because of the remarkable phenotype obtained by reducing the function of one of the predicted protease-encoding genes, CG4721 (Figs 6,7). The enrichment of peptidase-encoding genes in the eye-antennal disc may again reflect the fact that morphogenesis has already begun in the eye-antennal disc but not in the wing discs, and morphogenesis requires changes in cell-extracellular matrix contacts. Given the interesting phenotype obtained for the neprilysin encoded by CG4721, the functions of this group of genes may be worth pursuing.

### Insights into the eye gene network

In addition to the new genes identified in this study that are worth pursuing, our analysis provides interesting insights into the regulation of several genes already known for roles in eye development. We discuss a few of these below.

### Ey+N synergize to activate *ey* expression

Based on our transcriptomics data (Fig 4; Table S1), Ey and Notch are capable of activating *ey* transcription in the wing disc at levels that are significantly above the levels expressed from the *UAS-ey* transgene under control of *ap-Gal4*, suggesting that these factors synergize to activate *ey* expression during normal eye development. Accordingly, in the late third instar eye-antennal disc Notch activity levels are high ahead of the furrow where Ey expression is also highest [5], [20], [195], [196], [197]. This region contains cells that are fated to form eye but that have not yet started to differentiate. Instead, the cells in this region are actively proliferating under control of Notch activation along the dorsal-ventral midline [195], [196], [197].

Interestingly, Notch and Pax6 appear to have a similar relationship in vertebrates. In vertebrate retinal progenitor cells, Notch signaling maintains a proliferative state [198], [199], [200], [201], and Pax6 is required to maintain multipotency in proliferating cells [202]. Moreover, it has recently been shown that activating Notch in human embryonic stem cells (hESCs) induces Pax6, blocks mesodermal differentiation and promotes neuroectodermal commitment [203], [204], [205], [206]. It is not currently known whether this induction results from a synergism between Pax6 and Notch to maintain high levels of Pax6.

The potential synergism between Notch and Ey/Pax6 in regulating *ey/Pax6* expression is also interesting because it could reflect a general mechanism for cells to maintain multipotency while they are dividing under Notch control. For example, Notch is known to activate expression of transcription factors other than Ey/Pax6, depending on the cell lineage [204]. Thus, one mechanism that cells could use to maintain multipotency for a particular lineage as the cells are dividing, potentially in the face of competing information that might change cell fate, would be to have expression of the multipotency/lineage specification factor under control of both Notch and of itself. The synergism between Notch and the specification factor would result in much higher levels of the specification factor, locking in cell fate.

### Elav expression is controlled post-transcriptionally

Another surprise was the lack of response of *elav* transcription to misexpression of Ey or of Ey+signaling factors (Fig 4E), in spite of the fact that Elav protein expression is clearly elevated in these contexts (Fig 1). Levels of *elav* transcripts are in fact significantly higher in the eye control data versus the wing control data. This suggests that Elav expression may be regulated at both the transcriptional and post-transcriptional levels, with factors activated by Ey being responsible for primarily the post-transcriptional aspects of control.

Consistent with the idea that Elav may be regulated at the post-transcriptional level, *elav* transcripts are present in the wing control and at similar levels in all of the other wing disc libraries (38 unique reads per million in the wing control mRNAseq library, versus 278 reads per million for the eye control mRNAseq library; not shown). Therefore, at least small amounts of Elav protein are capable of being produced in wing discs at the stage we dissected. Ey may activate expression of a factor that e.g. increases the possibility of translation of the *elav* transcript, or that stabilizes the Elav protein.

At present we do not know the identity of this putative factor, nor have we identified additional genes important for eye development that are likely to be post-transcritptionally regulated. Moreover, GO clusters and terms associated with post-transcriptional regulation are exclusive to the “No Change” libraries (e.g. ubiquitin-protein ligase activity; response to dsRNA). Nevertheless, it is possible that the factor responsible for Elav post-transcriptional regulation is completely novel. In spite of our current ignorance about the mechanism, it is an important observation that Elav is likely to be post-transcriptionally regulated, not only for the insight into Elav regulation, but also because it highlights the fact that, as in other developmental contexts, not all genes are regulated at the transcriptional level.

### Ey+N downregulate *wg* expression

The synergism between Ey+N misexpression in the wing disc with respect to downregulation of *wg* levels (Fig 4F) was unexpected, particularly given that N signaling is known to activate Wg expression in the wing [85], [86], [87]. During eye development in *Drosophila*, Wg is expressed in cells adjacent to eye precursors, and it has previously been shown that high levels of Wg inhibit eye development and promote development of peripheral tissues, such as cuticle or pigment rim [88], [207], [208]. Likewise, whereas Wnt activity in some vertebrate CNS contexts drives cell proliferation [209], Wnt is also important in the vertebrate eye for development of peripheral eye structures, such as the ciliary body and the iris, and high levels of Wnt signaling can convert retinal cells to peripheral fates [210]. This example clearly illustrates the principle that, since signaling pathways like Notch are involved in essentially every aspect of development, the interaction between tissue-specific transcription factors and signaling pathways is crucial for allowing tissue-specific development.

### A gene with a novel role in eye development is highly upregulated by Ey+N

Perusing the lists of highly upregulated genes together with an *RNAi* screen of genes upregulated ≥3-fold versus the control wing disc has identified a number of interesting candidates for further study in the context of eye development. For one of these genes, *CG4721*, we have additional genetic data indicating a role in eye development. Thus, we fully expect that the transcriptome data we have generated will yield a wealth of genes with novel roles in *Drosophila* eye development.

The sequence of the *CG4721* gene indicates that it encodes a member of the neprilysin (M13) family of metalloendopeptidases, which cleave and thereby regulate the activity of a number of peptides in mammals. M13 peptidases are ectoenzymes that hydrolyze a large number of extracellular neuro- and signaling peptides. Among other substrates, mammalian M13 peptidases hydrolyze and regulate activity of (1) neuropeptides (e.g. tachykinins and enkephalins) [170]; (2) vasoconstrictors and vasodilators involved in blood pressure control (e.g. atrial natriuretic peptide and endothelin-1) [211]; and (3) bombesins and endothelin-1, which e.g. stimulate migration in prostate cancer cells [212], [213]. In addition, M13 peptidases hydrolyze and thereby reduce levels of the neurotoxic amyloid β-peptide in the context of Alzheimer's disease [214]. Finally, the M13 peptidases endothelin-converting-enzymes 1 and 2 also have developmental roles in the production of active endothelin, a peptide which functions as a signal in vertebrate neural crest cell development [215]. Although the protein encoded by the *CG4721* gene appears to lack the critical catalytic residues associated with metalloendopeptidase function, reduction of *CG4721* function via *RNAi* clearly affects early stages of eye development.

As mentioned above, *CG4721* is one of many genes encoding potential peptidases upregulated by the Ey+N combination. The question arises, why would *CG4721*, and potentially other peptidase genes, be a target of Ey+N in particular, as opposed to the e.g. Ey+Hh or Ey+Dpp. This question is especially relevant given that we have postulated that *CG4721* is involved in activating the Notch signaling pathway, rather than Notch activating *CG4721* (see Results). At present we do not have a clear answer to this. However, since Ey is required for initial expression of *ato*, and Notch signaling is a critical factor for proneural enhancement, it is possible that *CG4721* is part of a positive feedback loop designed to keep Notch activity high such that proneural enhancement can occur. Future work is required to determine whether this is true, and what the mechanisms are by which CG4721 affects Notch signaling.

### Summary

Our approach, which utilizes next-generation sequencing to examine the transcriptomes downstream of the eye specification factor Ey in concert with the Hh, Dpp or Notch signaling pathways, has identified new genes potentially involved in specification, differentiation and function of photoreceptor neurons, including a novel gene which we have demonstrated has a function in *Drosophila* eye development (*CG4721*). In addition, our data has indicated new directions to pursue in understanding the network between known genes important for eye development (e.g. synergism between Ey and Notch; *elav* post-transcriptional regulation). Future work will utilize *in silico* and molecular techniques, as well as the powerful genetics available in *Drosophila*, to further understanding of eye development.

## Materials and Methods

### Genetics

The following strains used in this study are available from the *Drosophila* Bloomington Stock Center and are described in FlyBase (http://flybase.org): *ap-Gal4*, *ey-Gal4*, *dan-Gal4*, *GMR-Gal4*, *mirr-Gal4*, *UAS-dpp, yw; Sp/CyO; dpp-lacZ{Pry+}, ry[506]/TM6,Ubx (BL-5528*), *A>F>G/FM6 ; UAS-GFP, hh-lacZ(27)/TM6B, w; 5*′ *3.5 ato-lacZ, w; 3*′ *ato-GFP* [25] (kind gift of Francesca Pignoni).

Other strains used: *UAS-ey* (on III) [20], *np2631-Gal4* (National Institute of Genetics, Japan), *sev-Gal4* [154], [216], *UAS-hh[M4]* [217], *UAS-N.ICN* [218], *UAS-CG4721^IR^ (51618)* (Vienna Drosophila *RNAi* Center), *hsflp; A>F>G, UAS-GFP* (on III) (courtesy of Dave O'Keefe) . Recombination was used to generate *UAS-ey, UAS-hh*; *UAS-ey, UAS-dpp*; and UAS-ey, UAS-N*; dan>CG4721/ 3′*ato-GFP (on III); dan>CG4721/ 5*′*ato-lacZ (on III); dan>CG4721/ dpp-lacZ; dan>CG4721/ hh-lacZ(27)* strains.

### Immunohistochemistry and Image Acquisition

Immunostainings were performed following standard protocols. We used the following primary antibodies: rat anti-Elav (1∶100), rat anti-DE-cad (1∶100), mouse anti-Eya (1∶20) and mouse anti-NICD from the Developmental Studies Hybridoma Bank (DSHB); guinea pig anti-Ato (1∶2000) [219] (kind gift of D. Marenda), and guinea pig anti-sens (1∶1000) (courtesy of H. Bellen). Primary antibodies were detected by incubating tissue in 1∶200 dilutions of the appropriate secondary antibodies (Jackson Immuno Research).

Images were obtained using an Axio Imager Z1 Fluorescent microscope with ApoTome and AxioCam MRm camera (Carl Zeiss MicroImaging, Inc) or a Leica TCS SC5 Laser Scanning Confocal Microscope available at the NMSU Core University Research Resource Laboratory (CURRL, NMSU). Confocal images are projections of multiple sections obtained using ImageJ.

### Scanning Electron Microscopy (SEM)

SEM images of adult eyes were generated as previously described [154], [220].

### RNA purification and Illumina Sequencing

Approximately150 eye or wing discs were dissected from each of the genotypes: eye control (*ap-Gal4*); wing control (*ap-Gal4*); ap>ey; ap>hh; ap>dpp; ap>N*; ap>ey,hh; ap>ey,dpp; ap>ey,N*) and total RNA purified. Dissections were performed in 1X PBS and discs from each genotype were pooled in single 1.5 ml tubes containing 300 µl of tissue lysis buffer. Samples were kept at −80°C until RNA purification or purified immediately after dissection. Each sample tube was pipetted up and down to mix and the lysate homogenized by passing it through a sterile 20-gauge needle at least 10 times. Total RNA was purified from each sample lysate using an RNeasy Miniprep Kit (Qiagen) per the manufacturers' protocol and stored at −80°C.

Total RNA was quantified using a NanoDrop® ND-1000 UV spectrophotometer (NanoDrop Technologies). 5–14 µg of total RNA was purified from each genotype. RNA quality was evaluated by agarose gel electrophoresis. RNA integrity was further assessed using an Agilent 2100 Bioanalyzer (Agilent Inc., Santa Clara, CA). PolyA+ RNA was isolated from total RNA by two rounds of oligo-dT selection (Invitrogen Inc., Santa Clara, CA) and mRNASeq libraries were made and sequenced on an Illumina Genome Analyzer IIx (GAIIx) platform according to manufacturer instructions. Library preparation and sequencing were performed at NCGR.

### Read alignment

Reads generated from Illumina sequencing runs were aligned to the *Drosophila* Genomic Sequence Release 5 (Berkeley *Drosophila* Genome Project) and to the All-Transcript Sequence Release 5.21 (FlyBase) using the GSNAP algorithm and the Alpheus software system at NCGR. GSNAP is a modified version of GMAP [221] that handles short reads [57] and is therefore suitable for Illumina read alignments. Alignment parameters and read normalization were performed as described in MUDGE *et al.* [61].

### Agilent whole genome array

Total RNA, PolyA+ RNA and cDNA preparations for Agilent array analyses were performed as for Illumina sequencing. Agilent array analysis was performed at the Nationwide Children's Hospital Biomedical Genomics Core (NCHBGC) facility in Columbus, Ohio. At the NCHBGC facility, the concentration of RNA samples was determined using the NanoDrop® ND-1000 UV Spectrophotometer. The integrity of RNA samples was determined using an Agilent 2100 Bioanalyzer, Lab-On-A-Chip Agilent 6000 Series II chip. All samples met or exceeded the quality control (QC) cutoff established at the NCHBGC.

All 36 (4 biological replicates X 9 libraries) labeled cDNA libraries generated from purified mRNAs were hybridized to *D. melanogaster* whole genome 4X44K Agilent expression arrays, per the manufacturers’ protocol. A one-color array procedure was performed by hybridizing slides overnight, washing and scanning with an Agilent G2505C Microarray Scanner. Probe intensities from arrays were extracted from image data using Agilent Feature Extraction 10.5 (FE) and normalization and data processing were performed as described below.

### Bioinformatics and Statistical Analyses

Raw intensity values extracted from array image data sets were imported into the statistical computing and graphics software environment “R”. “R” was used for all data input, diagnostic plots and normalization. Analyses were performed using customized scripts developed in-house at the NCHBGC, which utilize several Bioconductor packages [222].

Median intensity values were extracted from the FE data files for each probe on the array. It has been demonstrated that background subtraction of raw intensities from Agilent arrays can introduce variability into the data, especially at the low intensity end [223]; therefore we did not perform background correction of the intensity data. Present, marginal or absent calls were made using methodologies developed by the NCHBGC and taking into account intensity values determined by the Agilent FE program together with intensity values for the negative controls present in the arrays. Intensity normalization was performed using Quantile Normalization [62].

The relative differences in gene expression were calculated using the array statistical tool, Significance Analyses of Microarrays (SAM) [63]. SAM identified statistically significant genes by carrying out gene specific t-tests and measured the strength of the relationship between gene expression and the response variable. It also corrected for experimental errors by calculating the false discovery rate (FDR). An FDR of 10–20% was used as an acceptable cut-off range.

Illumina read frequencies and Array hybridization signals were log_2_ transformed prior to analyzing expression differences between samples. The unsupervised principal component analyses (by Pearson product-moment correlation), univariate kernel density distribution curves, box plot distributions, pair-wise sample correlations, Pearson product-moment correlations and 2-way hierarchical clustering were all performed using JMP Genomics version 4.1 (SAS Institute, Cary, NC). We also used JMP Genomics to analyze the distribution of read frequencies along the genome and also to determine statistically significant differences in fold changes between libraries.

Candidate genes were functionally annotated and grouped into different categories using the DAVID (Database for Annotation, Visualization and Integrated Discovery) Bioinformatic tool at NIH, according to Gene Ontology Biological Process and Molecular Function terms. GOStat from Gene Ontology was used to confirm annotations and validate gene groupings. GO terms of similar Biological or Molecular processes were grouped together into categories under a label that best summarized all GO terms.

## Supporting Information

Figure S1 Great similarity exists between mRNASeq libraries. Pair-wise correlation coefficients (R^2^) between libraries were very high ranging from 0.92 to 0.99.
(TIF)

Figure S2 Based on overlaid one-way kernel density distribution curves (A), box plots (B) and relative log expression plots, array data are of high quality.
(TIF)

Figure S3 Great similarity exists between array libraries. Pair-wise correlation coefficients (R^2^) between libraries ranged between 0.94 and 0.99.
(TIF)

Figure S4 Array and mRNAseq data correlate weakly. Scatterplot of average log_2_-transformed fold change intensities for array plotted against log_2_-transformed fold change reads per million (relative to wing control).
(TIF)

Figure S5 (A) *3*′*ato-GFP* is expressed ahead of and within the furrow in otherwise wild-type eye-antennal discs. (B) *3*′*ato-GFP* is expressed in a broader band in *dan>CG4721^IR^/3*′*ato-GFP* discs. (C) *5*′*ato-lacZ* is expressed in ocellar precursors (white arrow) and posterior to the furrow (black arrow) in otherwise wild-type eye-antennal discs. (D) *5*′*ato-lacZ* is expression in ocellar precursors (white arrow), but not posterior to the furrow in *dan>CG4721^IR^ /5*′ *ato-lacZ* discs.
(TIF)

Table S1 Eye genes are upregulated and wing genes are downregulated when Ey+signaling factors are misexpressed in the wing. Fold change values for the indicated genes for both array and mRNASeq. **Array data**: pink and green shading indicate statistically significant upregulation and downregulation, respectively, based on the SAM statistical analysis as described in the text. mRNASeq data: blue shading indicates >1.7X upregulation; orange shading indicates >2.5X downregulation; yellow shading indicates 0 reads aligned to a particular gene, with fold changes determined using 0.5 to avoid division by 0.
(XLSX)

Table S2 More eye-specific genes are expressed at ≥3-fold versus the wing when Ey+signaling factors are misexpressed compared to when Ey alone is misexpressed. Genes in bold were found in clusters obtained in the DAVID analysis (see tables S3–S5).
(XLS)

Table S3 Summary of DAVID analysis of gene ontology terms for biological and molecular function. The number of genes entered into the DAVID software, and the number that were included in at least one cluster are shown in the headings.
(XLSX)

Table S4 Raw data for DAVID analysis of eye control, *ap>ey*, *ap>ey,hh*, *ap>ey,dpp*, *ap>ey,N* and wing control libraries.
(XLSX)

Table S5 Raw data for DAVID analysis of lists of genes with no change in expression in eye control vs. wing control libraries.
(XLSX)

Table S6 List of a subset of genes in DAVID clusters associated with neuronal differentiation.
(XLSX)

Table S7 List of a subset of genes in DAVID clusters associated with photoreceptor/neuronal function.
(XLSX)

Table S8 List of the eye/photoreceptor specification factors found in the “eye/photoreceptor development” DAVID clusters in different libraries.
(XLSX)

Table S9 List of a subset of genes in “peptidase” DAVID clusters.
(XLSX)
