# Supplementary figures and images for: Analysis of the Transcriptomes Downstream of Eyeless and the Hedgehog, Decapentaplegic and Notch Signaling Pathways in Drosophila melanogaster
Source: PLoS One. 2012 Aug 31;7(8):e44583. doi: 10.1371/journal.pone.0044583 (PMC3432130; doi:10.1371/journal.pone.0044583)

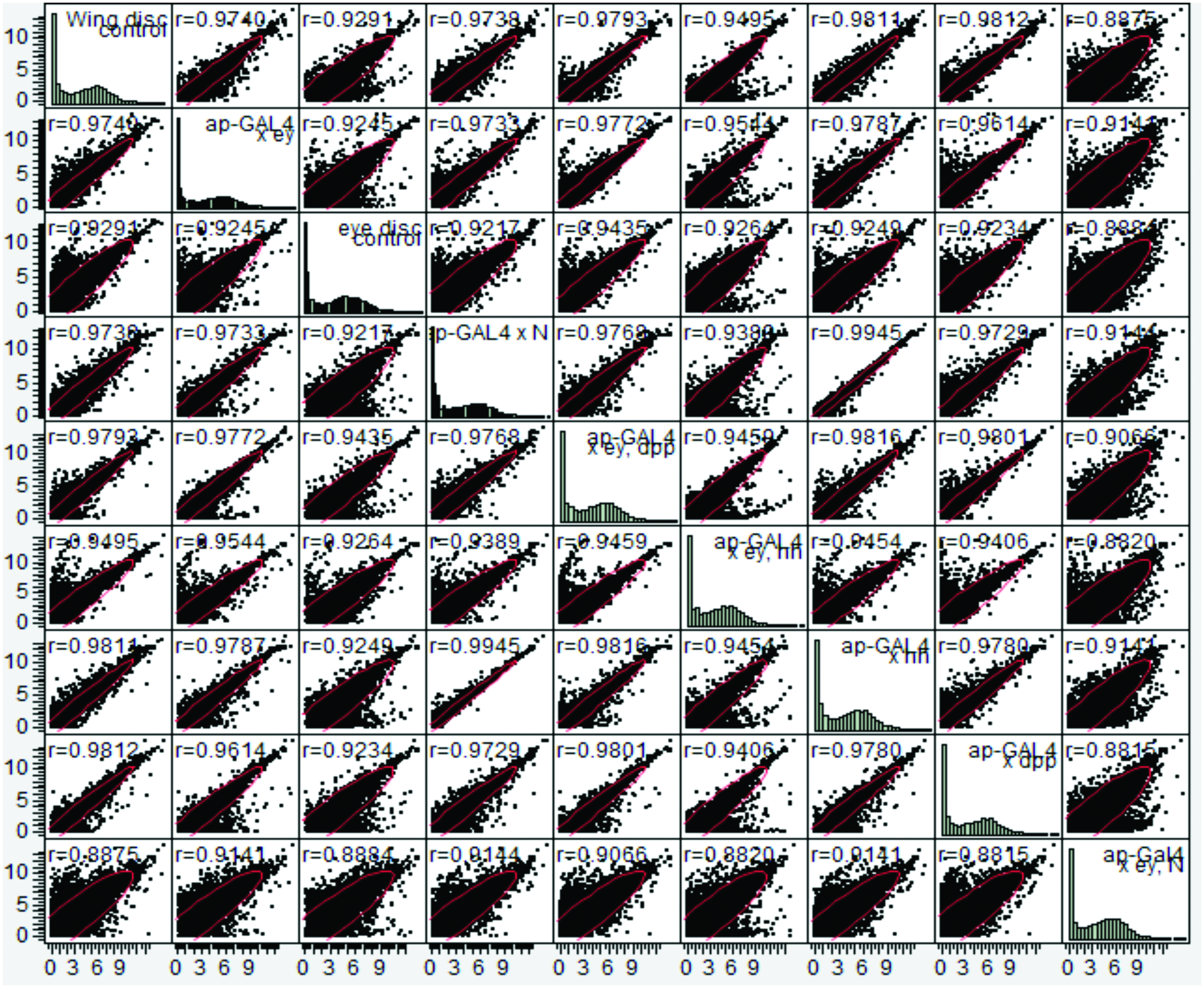

Supplement: Figure S1 — Great similarity exists between mRNASeq libraries. Pair-wise correlation coefficients (R2) between libraries were very high ranging from 0.92 to 0.99. (TIF) [file pone.0044583.s001.tif]

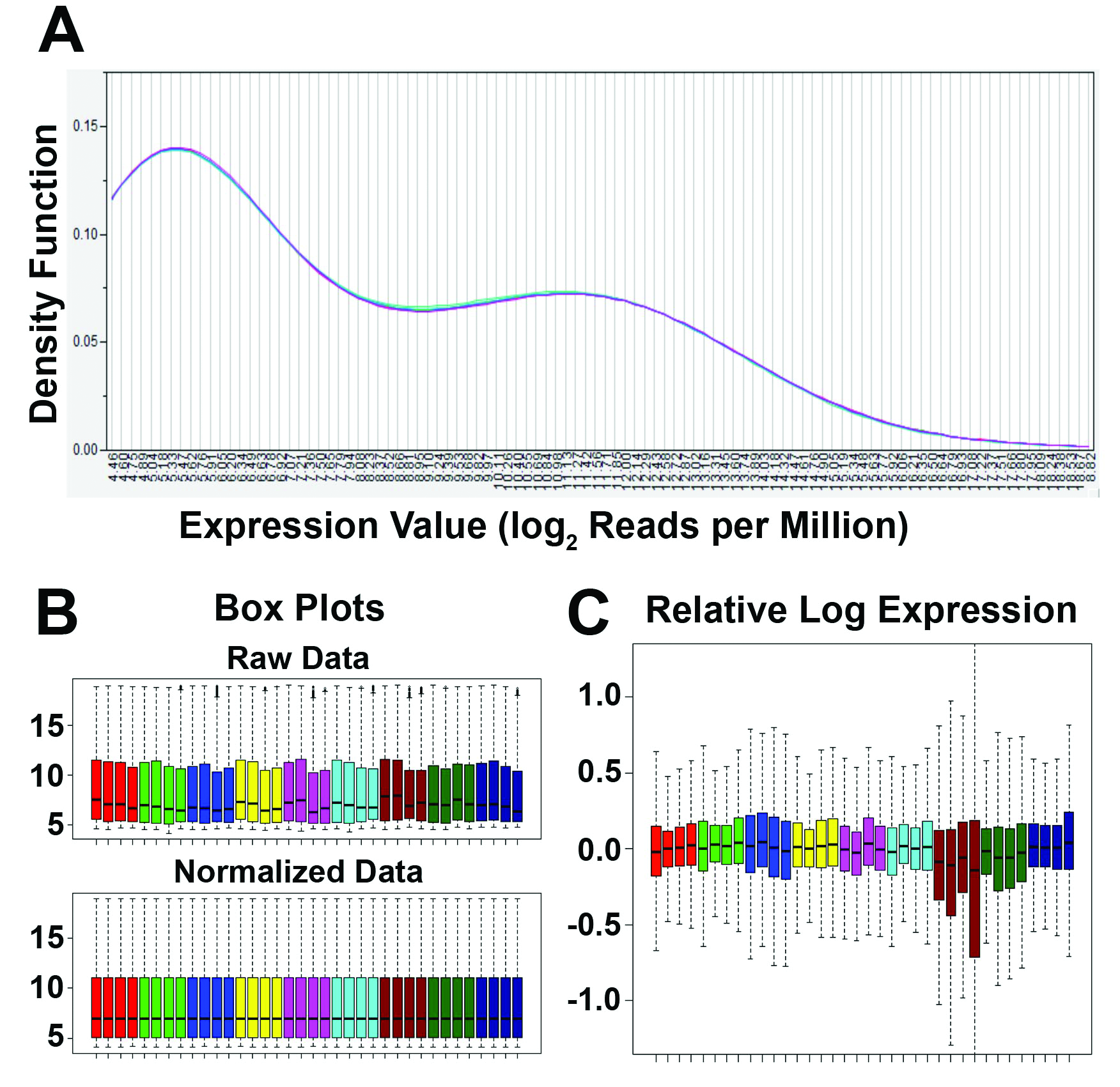

Supplement: Figure S2 — Based on overlaid one-way kernel density distribution curves (A), box plots (B) and relative log expression plots, array data are of high quality. (TIF) [file pone.0044583.s002.tif]

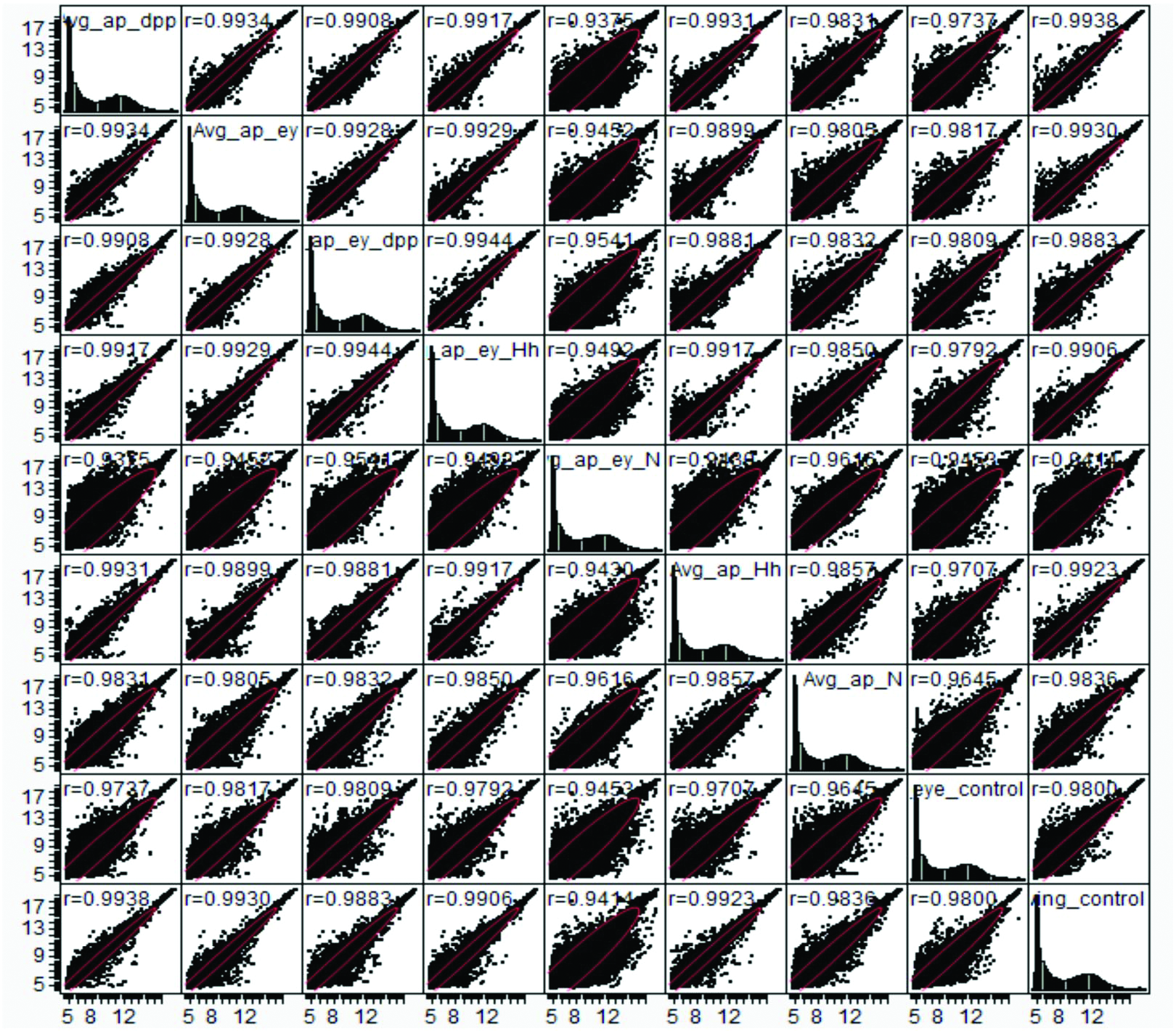

Supplement: Figure S3 — Great similarity exists between array libraries. Pair-wise correlation coefficients (R2) between libraries ranged between 0.94 and 0.99. (TIF) [file pone.0044583.s003.tif]

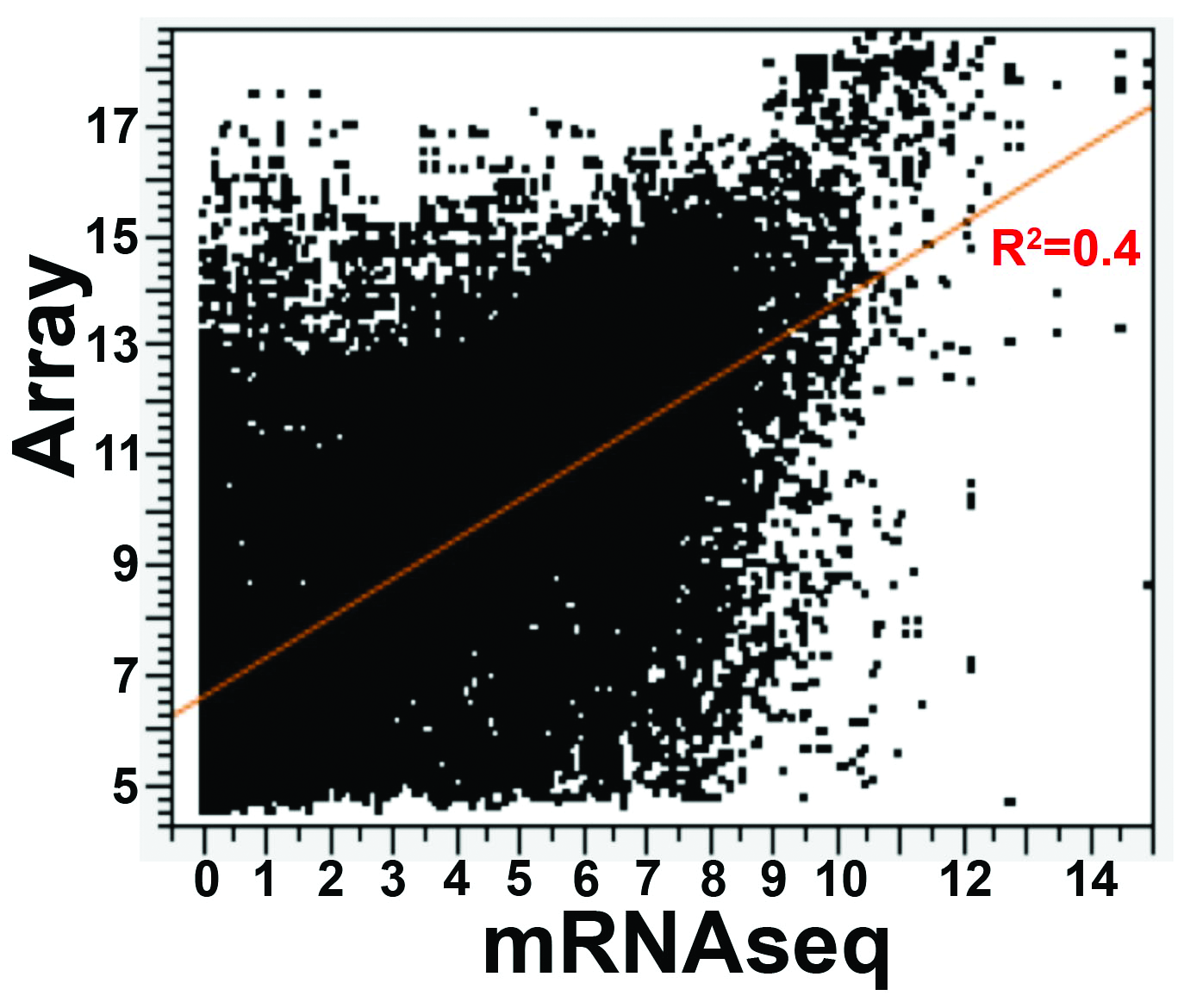

Supplement: Figure S4 — Array and mRNAseq data correlate weakly. Scatterplot of average log2-transformed fold change intensities for array plotted against log2-transformed fold change reads per million (relative to wing control). (TIF) [file pone.0044583.s004.tif]

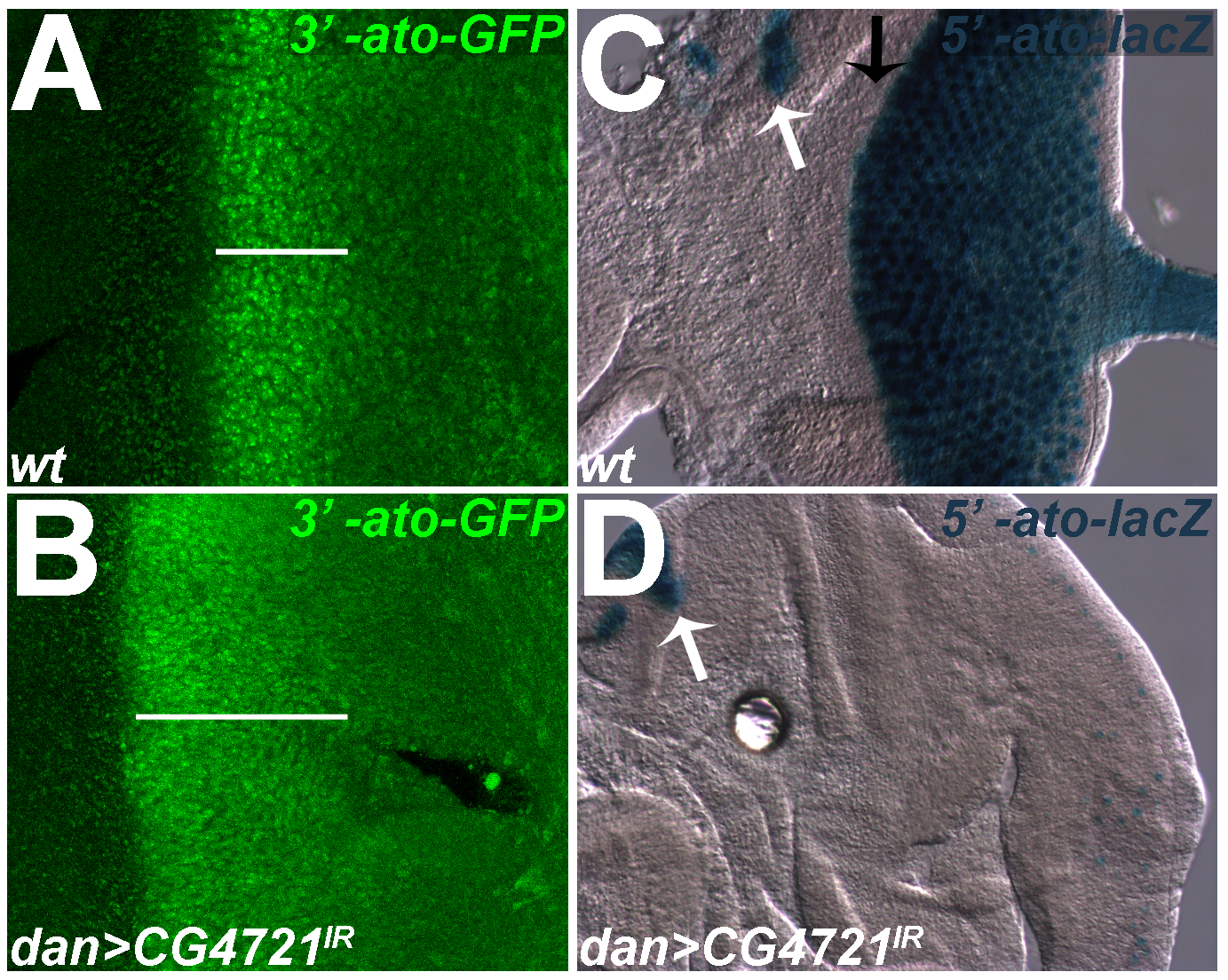

Supplement: Figure S5 — (A) 3′ato-GFP is expressed ahead of and within the furrow in otherwise wild-type eye-antennal discs. (B) 3′ato-GFP is expressed in a broader band in dan>CG4721IR/3′ato-GFP discs. (C) 5′ato-lacZ is expressed in ocellar precursors (white arrow) and posterior to the furrow (black arrow) in otherwise wild-type eye-antennal discs. (D) 5′ato-lacZ is expression in ocellar precursors (white arrow), but not posterior to the furrow in dan>CG4721IR /5′ ato-lacZ discs. (TIF) [file pone.0044583.s005.tif]
